# Shared genetic architecture between obesity and psychiatric disorders reveals comorbid etiology and therapeutic targets

**DOI:** 10.1093/lifemedi/lnag015

**Published:** 2026-04-22

**Authors:** Dong Liu, Chun Dou, Chaojie Ye, Mingling Chen, Lijie Kong, Zheng Zhu, Jie Zheng, Min Xu, Yu Xu, Mian Li, Zhiyun Zhao, Jieli Lu, Yuhong Chen, Zhongshang Yuan, Guang Ning, Weiqing Wang, Yufang Bi, Tiange Wang

**Affiliations:** Department of Endocrine and Metabolic Diseases, Shanghai Institute of Endocrine and Metabolic Diseases, Ruijin Hospital, Shanghai Jiao Tong University School of Medicine, Shanghai 200025, China; Shanghai National Clinical Research Center for Metabolic Diseases, Key Laboratory for Endocrine and Metabolic Diseases of the National Health Commission of the PR China, Shanghai Key Laboratory for Endocrine Tumor, Ruijin Hospital, Shanghai Jiao Tong University School of Medicine, Shanghai 200025, China; Department of Endocrine and Metabolic Diseases, Shanghai Institute of Endocrine and Metabolic Diseases, Ruijin Hospital, Shanghai Jiao Tong University School of Medicine, Shanghai 200025, China; Shanghai National Clinical Research Center for Metabolic Diseases, Key Laboratory for Endocrine and Metabolic Diseases of the National Health Commission of the PR China, Shanghai Key Laboratory for Endocrine Tumor, Ruijin Hospital, Shanghai Jiao Tong University School of Medicine, Shanghai 200025, China; Department of Endocrine and Metabolic Diseases, Shanghai Institute of Endocrine and Metabolic Diseases, Ruijin Hospital, Shanghai Jiao Tong University School of Medicine, Shanghai 200025, China; Shanghai National Clinical Research Center for Metabolic Diseases, Key Laboratory for Endocrine and Metabolic Diseases of the National Health Commission of the PR China, Shanghai Key Laboratory for Endocrine Tumor, Ruijin Hospital, Shanghai Jiao Tong University School of Medicine, Shanghai 200025, China; Department of Endocrine and Metabolic Diseases, Shanghai Institute of Endocrine and Metabolic Diseases, Ruijin Hospital, Shanghai Jiao Tong University School of Medicine, Shanghai 200025, China; Shanghai National Clinical Research Center for Metabolic Diseases, Key Laboratory for Endocrine and Metabolic Diseases of the National Health Commission of the PR China, Shanghai Key Laboratory for Endocrine Tumor, Ruijin Hospital, Shanghai Jiao Tong University School of Medicine, Shanghai 200025, China; Department of Endocrine and Metabolic Diseases, Shanghai Institute of Endocrine and Metabolic Diseases, Ruijin Hospital, Shanghai Jiao Tong University School of Medicine, Shanghai 200025, China; Shanghai National Clinical Research Center for Metabolic Diseases, Key Laboratory for Endocrine and Metabolic Diseases of the National Health Commission of the PR China, Shanghai Key Laboratory for Endocrine Tumor, Ruijin Hospital, Shanghai Jiao Tong University School of Medicine, Shanghai 200025, China; Department of Endocrine and Metabolic Diseases, Shanghai Institute of Endocrine and Metabolic Diseases, Ruijin Hospital, Shanghai Jiao Tong University School of Medicine, Shanghai 200025, China; Shanghai National Clinical Research Center for Metabolic Diseases, Key Laboratory for Endocrine and Metabolic Diseases of the National Health Commission of the PR China, Shanghai Key Laboratory for Endocrine Tumor, Ruijin Hospital, Shanghai Jiao Tong University School of Medicine, Shanghai 200025, China; Department of Endocrine and Metabolic Diseases, Shanghai Institute of Endocrine and Metabolic Diseases, Ruijin Hospital, Shanghai Jiao Tong University School of Medicine, Shanghai 200025, China; Shanghai National Clinical Research Center for Metabolic Diseases, Key Laboratory for Endocrine and Metabolic Diseases of the National Health Commission of the PR China, Shanghai Key Laboratory for Endocrine Tumor, Ruijin Hospital, Shanghai Jiao Tong University School of Medicine, Shanghai 200025, China; Department of Endocrine and Metabolic Diseases, Shanghai Institute of Endocrine and Metabolic Diseases, Ruijin Hospital, Shanghai Jiao Tong University School of Medicine, Shanghai 200025, China; Shanghai National Clinical Research Center for Metabolic Diseases, Key Laboratory for Endocrine and Metabolic Diseases of the National Health Commission of the PR China, Shanghai Key Laboratory for Endocrine Tumor, Ruijin Hospital, Shanghai Jiao Tong University School of Medicine, Shanghai 200025, China; Department of Endocrine and Metabolic Diseases, Shanghai Institute of Endocrine and Metabolic Diseases, Ruijin Hospital, Shanghai Jiao Tong University School of Medicine, Shanghai 200025, China; Shanghai National Clinical Research Center for Metabolic Diseases, Key Laboratory for Endocrine and Metabolic Diseases of the National Health Commission of the PR China, Shanghai Key Laboratory for Endocrine Tumor, Ruijin Hospital, Shanghai Jiao Tong University School of Medicine, Shanghai 200025, China; Department of Endocrine and Metabolic Diseases, Shanghai Institute of Endocrine and Metabolic Diseases, Ruijin Hospital, Shanghai Jiao Tong University School of Medicine, Shanghai 200025, China; Shanghai National Clinical Research Center for Metabolic Diseases, Key Laboratory for Endocrine and Metabolic Diseases of the National Health Commission of the PR China, Shanghai Key Laboratory for Endocrine Tumor, Ruijin Hospital, Shanghai Jiao Tong University School of Medicine, Shanghai 200025, China; Department of Endocrine and Metabolic Diseases, Shanghai Institute of Endocrine and Metabolic Diseases, Ruijin Hospital, Shanghai Jiao Tong University School of Medicine, Shanghai 200025, China; Shanghai National Clinical Research Center for Metabolic Diseases, Key Laboratory for Endocrine and Metabolic Diseases of the National Health Commission of the PR China, Shanghai Key Laboratory for Endocrine Tumor, Ruijin Hospital, Shanghai Jiao Tong University School of Medicine, Shanghai 200025, China; Department of Endocrine and Metabolic Diseases, Shanghai Institute of Endocrine and Metabolic Diseases, Ruijin Hospital, Shanghai Jiao Tong University School of Medicine, Shanghai 200025, China; Shanghai National Clinical Research Center for Metabolic Diseases, Key Laboratory for Endocrine and Metabolic Diseases of the National Health Commission of the PR China, Shanghai Key Laboratory for Endocrine Tumor, Ruijin Hospital, Shanghai Jiao Tong University School of Medicine, Shanghai 200025, China; Department of Endocrine and Metabolic Diseases, Shanghai Institute of Endocrine and Metabolic Diseases, Ruijin Hospital, Shanghai Jiao Tong University School of Medicine, Shanghai 200025, China; Shanghai National Clinical Research Center for Metabolic Diseases, Key Laboratory for Endocrine and Metabolic Diseases of the National Health Commission of the PR China, Shanghai Key Laboratory for Endocrine Tumor, Ruijin Hospital, Shanghai Jiao Tong University School of Medicine, Shanghai 200025, China; Department of Biostatistics, School of Public Health, Cheeloo College of Medicine, Institute for Medical Dataology, Shandong University, Jinan, Shandong 250012, China; Department of Endocrine and Metabolic Diseases, Shanghai Institute of Endocrine and Metabolic Diseases, Ruijin Hospital, Shanghai Jiao Tong University School of Medicine, Shanghai 200025, China; Shanghai National Clinical Research Center for Metabolic Diseases, Key Laboratory for Endocrine and Metabolic Diseases of the National Health Commission of the PR China, Shanghai Key Laboratory for Endocrine Tumor, Ruijin Hospital, Shanghai Jiao Tong University School of Medicine, Shanghai 200025, China; Department of Endocrine and Metabolic Diseases, Shanghai Institute of Endocrine and Metabolic Diseases, Ruijin Hospital, Shanghai Jiao Tong University School of Medicine, Shanghai 200025, China; Shanghai National Clinical Research Center for Metabolic Diseases, Key Laboratory for Endocrine and Metabolic Diseases of the National Health Commission of the PR China, Shanghai Key Laboratory for Endocrine Tumor, Ruijin Hospital, Shanghai Jiao Tong University School of Medicine, Shanghai 200025, China; Department of Endocrine and Metabolic Diseases, Shanghai Institute of Endocrine and Metabolic Diseases, Ruijin Hospital, Shanghai Jiao Tong University School of Medicine, Shanghai 200025, China; Shanghai National Clinical Research Center for Metabolic Diseases, Key Laboratory for Endocrine and Metabolic Diseases of the National Health Commission of the PR China, Shanghai Key Laboratory for Endocrine Tumor, Ruijin Hospital, Shanghai Jiao Tong University School of Medicine, Shanghai 200025, China; Department of Endocrine and Metabolic Diseases, Shanghai Institute of Endocrine and Metabolic Diseases, Ruijin Hospital, Shanghai Jiao Tong University School of Medicine, Shanghai 200025, China; Shanghai National Clinical Research Center for Metabolic Diseases, Key Laboratory for Endocrine and Metabolic Diseases of the National Health Commission of the PR China, Shanghai Key Laboratory for Endocrine Tumor, Ruijin Hospital, Shanghai Jiao Tong University School of Medicine, Shanghai 200025, China

## Abstract

Integrated genomic evidence on shared genetic architecture between obesity and psychiatric disorders remains limited. This work utilized multi-level genomic analytic approaches to identify pleiotropic loci, variants, and genes between 14 adiposity traits and 7 psychiatric disorders, including genetic correlation and bidirectional causality as well as gene expression, functional pathway, and druggability, using genome-wide association study data from up to 806,834 individuals of European descent. Based on 67 genetically correlated trait pairs established between the two groups, we identified 17 causal shared genes across 26 tissues, which were enriched in neurodevelopment, neuronal function, cellular transport, and developmental biology. Of these, *NEGR1*, *CTNNB1*, *TAOK2*, and *RTN4RL1* were located in the druggable genome, and *CTNNB1* and *NT5C2* were clinically actionable. Mendelian randomization further supported extensive bidirectional causal associations. These findings provide robust evidence for a shared genetic etiology between adiposity and psychiatric disorders, underscoring mechanistic links and prioritizing actionable targets for comorbidity intervention.

## Introduction

The worldwide prevalence of overweight and obesity has doubled in the past three decades [1]. Concomitantly, the prevalence of psychiatric disorders such as depression, anxiety disorder (ANX), and schizophrenia (SCZ) is rising steadily [2]. Recent observational evidence has associated obesity with depression [3], and genetic evidence by Mendelian randomization (MR) has suggested causal associations of body mass index (BMI) with major depressive disorder (MDD), attention deficit hyperactivity disorder (ADHD), and SCZ [4, 5]. Although the relationship between obesity and psychiatric disorders is complex, their shared risk factors (e.g. unhealthy eating habits, physical inactivity, and disrupted sleeping patterns) and pathogenetic pathways (e.g. inflammation and stress) imply potential shared etiology between the two [6, 7].

Thus far, nuanced evidence on shared genetic architecture between obesity and psychiatric disorders is limited, hindering the understanding of their interacting mechanisms and the translation of current evidence into clinical applications for disease prevention and control. At least two uncertainties need to be clarified. First, although previous genetic analyses identified shared genes between BMI and SCZ [5], given the heterogeneity of adiposity [8], BMI may not fully represent adiposity and capture the shared signaling between adiposity and psychiatric disorders. Alternatively, taking advantage of magnetic resonance imaging (MRI) or body composition analyzer data and machine learning methods, precise and specific adiposity traits such as body fat percentage (BF%) and fat distribution of limbs, trunks, and organs have been constructed and can be utilized to generate comprehensive and accurate evidence for adiposity–psychiatry cross-talk [9–13]. Second, whether there are pleiotropic, druggable genetic targets between adiposity traits and psychiatric disorders is unclear, which can inform potential side effects or pleiotropic benefits of medications confronting the two groups of disorders. For example, raising concerns about whether weight-loss medications, glucagon-like peptide-1 receptor agonists, modify rates of psychiatric conditions [14, 15], as well as the additional impact of antidepressants on body weight [16]. Therefore, pleiotropic, druggable genetic targets for adiposity traits and psychiatric disorders are warranted for developing effective and personalized treatments, enhancing our understanding of the underlying biology, and improving holistic outcomes.

To this end, this study applied multi-level genomic analyses to delineate the genetic correlations and bidirectional causal associations between adiposity traits and psychiatric disorders using the largest genome-wide association study (GWAS) summary statistics to date from individuals of European descent. Under the analytical framework of the shared genetic architecture, we further identified shared genetic variants and genes between 14 adiposity traits and 7 psychiatric disorders across 26 relevant tissues, assessed the function and druggability of the identified causal genes, and explored the potential druggability for comorbidity therapeutic intervention.

## Results

### Study overview

**Figure 1.**
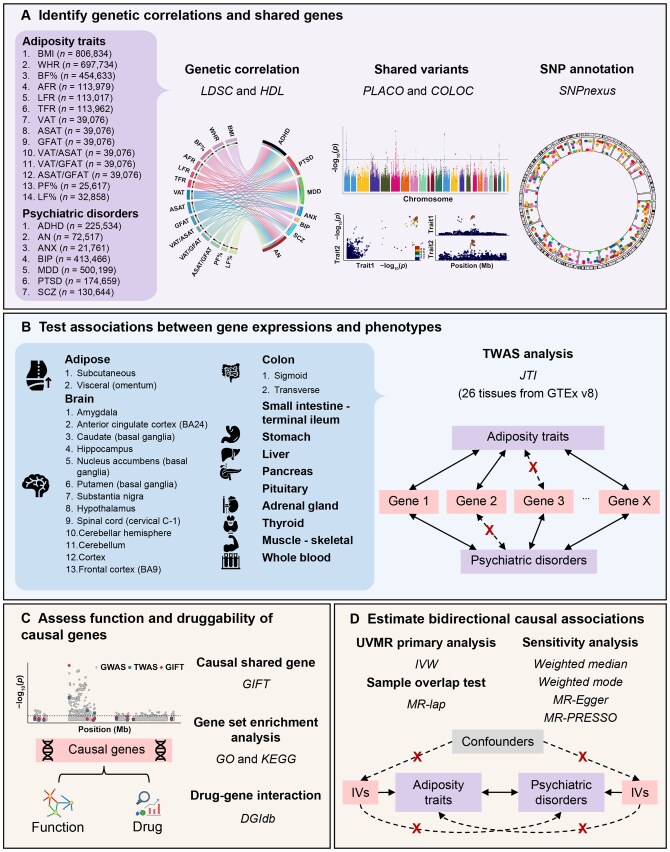
Schematic diagram of this multi-level genomic study. (A) Pairs of adiposity traits and psychiatric disorders with significant genetic correlation in LDSC or HDL analysis were defined as linkage pairs. Candidate shared variants between each linkage pair were identified by PLACO and COLOC analyses. Based on the shared variants, candidate shared genes were annotated using SNPnexus. (B) Shared genes with genetically predicted expression levels were significantly associated with both adiposity traits and psychiatric disorders in at least one tissue using JTI TWAS analysis. (C) Causal genes were filtered from the shared genes by using the conditional TWAS analysis. Gene set enrichment analysis and DGIdb were applied to the causal shared genes. (D) Bidirectional UVMR analysis was performed to assess the causal associations between adiposity traits and psychiatric disorders. The elements in the schematic diagram were designed using resources from Flaticon.com. Abbreviations: ADHD, attention deficit hyperactivity disorder; AFR, arms–arm fat ratio; AN, anorexia nervosa; ANX, anxiety; ASAT, abdominal subcutaneous adipose tissue; BF%, body fat percentage; BIP, bipolar disorder; BMI, body mass index; COLOC, Bayesian colocalization test; DGIdb, Drug Gene Interaction database; GIFT, gene-based integrative fine-mapping through conditional TWAS; GFAT, gluteofemoral adipose tissue; GO, Gene Ontology; GWAS, genome-wide association study; HDL, high-definition likelihood; IVs, instrumental variants; IVW, inverse-variance weighted; JTI, joint-tissue imputation; KEGG, Kyoto Encyclopedia of Genes and Genomes; LF%, liver fat percentage; LFR, legs–leg fat ratio; LDSC, linkage disequilibrium score regression; MDD, major depressive disorder; MR-PRESSO, Mendelian randomization pleiotropy residual sum and outlier; PF%, pancreas fat percentage; PLACO, pleiotropic analysis under composite null hypothesis; PTSD, post-traumatic stress disorder; SCZ, schizophrenia; SNP, single nucleotide polymorphism; TFR, trunk–trunk fat ratio; TWAS, transcriptome-wide association study; UVMR, univariable MR; VAT, visceral adipose tissue; WHR, waist-to-hip ratio. The elements in the graphic summary were designed using resources from Flaticon.com.

Figure 1 shows the study design and analytical framework of this analysis. Table S1 summarizes the detailed information on GWAS summary statistics of 14 adiposity traits and 7 psychiatric disorders.

### Genetic correlations and shared genes

The linkage disequilibrium score regression (LDSC) and high-definition likelihood (HDL) analyses established 67 genetically correlated linkage pairs between 14 adiposity traits and 7 psychiatric disorders (Tables S2 and S3). Besides, 57 and 41 genetically correlated linkage pairs were identified for women’s and men’s adiposity traits with 7 psychiatric disorders, respectively (Fig. S1). Adiposity traits were positively correlated with ADHD, post-traumatic stress disorder (PTSD), and MDD but inversely correlated with anorexia nervosa (AN) and SCZ, consistently for general adiposity traits as well as women’s and men’s adiposity traits (Tables S2 and S3). By contrast, the leg–leg fat ratio (LFR), for both the general trait (*r_g_* = 0.13, standard error [SE] = 0.03) and men’s trait (0.34, 0.03), was positively correlated with ADHD, but for women’s trait, it was inversely correlated with ADHD (0.07, 0.02).

**Figure 2.**
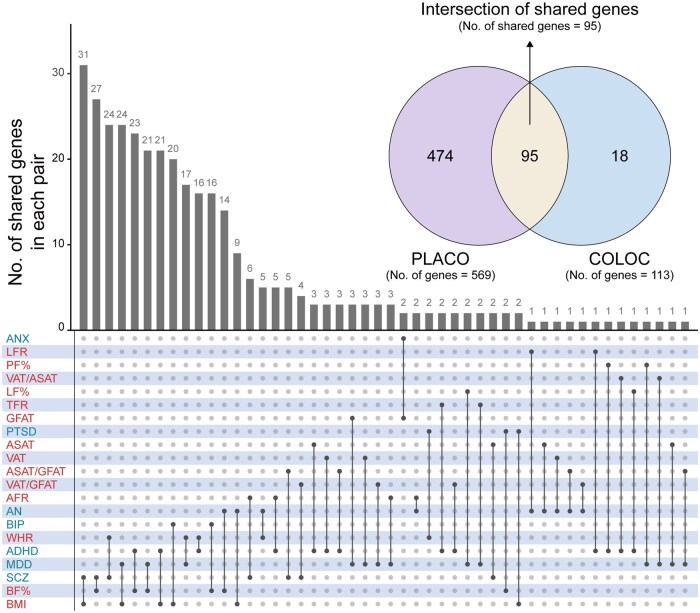
Shared genes between adiposity traits and psychiatric disorders identified by PLACO and COLOC. The bar chart indicates the number of shared genes in the intersection of PLACO and COLOC analyses. The linked points in each column indicate the specific adiposity traits and psychiatric disorders in each linkage pair, with adiposity traits marked in red and psychiatric disorders in blue. The Venn diagram shows the shared genes identified by PLACO and COLOC and their intersection. Abbreviations: ADHD, attention deficit hyperactivity disorder; AFR, arms–arm fat ratio; AN, anorexia nervosa; ANX, anxiety; ASAT, abdominal subcutaneous adipose tissue; BF%, body fat percentage; BIP, bipolar disorder; BMI, body mass index; COLOC, Bayesian colocalization test; GFAT, gluteofemoral adipose tissue; LF%, liver fat percentage; LFR, legs–leg fat ratio; MDD, major depressive disorder; PF%, pancreas fat percentage; PLACO, pleiotropic analysis under composite null hypothesis; PTSD, post-traumatic stress disorder; SCZ, schizophrenia; TFR, trunk–trunk fat ratio; VAT, visceral adipose tissue; WHR, waist-to-hip ratio.

In each linkage pair of adiposity traits and psychiatric disorders, pleiotropic analysis under composite null hypothesis (PLACO) analysis identified 846 shared variants, which were annotated to 569 genes, and colocalization (COLOC) analysis identified 139 shared variants and 113 shared genes (Fig. 2, Tables S4 and S5). With regard to the number of linkage pairs, the top four shared genes were *RP11-6N13.1*, *MAD1L1*, *BDNF-AS*, and *RP11-436D23.1*, which were identified in 22, 14, 10, and 10 linkage pairs, respectively. Both PLACO and COLOC analyses identified 95 shared genes between adiposity traits and psychiatric disorders (Table S6). The intersection of shared genes between adiposity and psychiatric disorders identified by PLACO and COLOC was 72 and 45 for women’s and men’s adiposity traits, respectively (Fig. S2, Tables S7 and S8).

### Associations between tissue-specific genetically predicted gene expression levels and traits

**Figure 3.**
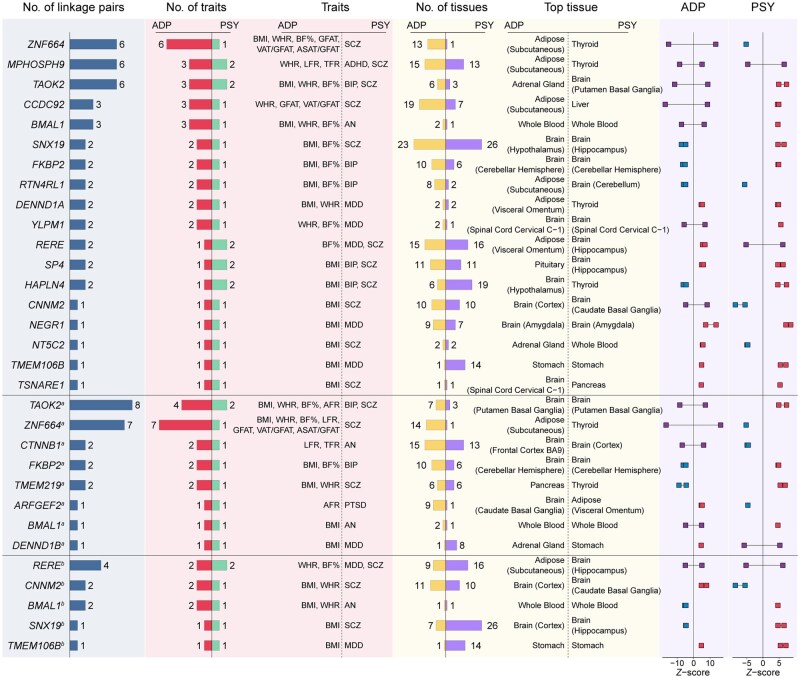
Shared genes between adiposity traits and psychiatric disorders identified by JTI TWAS in specific tissues. The first column shows the number of linkage pairs between adiposity traits and psychiatric disorders related to specific shared genes. The second column shows the number of both groups of traits in each linkage pair related to specific shared genes, and the third column lists the corresponding traits of both groups. The fourth and fifth columns show the number of tissues and the top tissues of each linkage pair related to specific shared genes. The sixth and seventh columns show the range of *Z*-scores of the associations between tissue-specific genetically predicted gene expression and traits (the sixth column: adiposity traits; the seventh column: psychiatric disorders) related to specific shared genes. The red, blue, or purple squares in columns six and seven indicate that the range of *Z*-scores are all positive, all negative, or inconsistent, respectively. ‘a’ Indicates shared genes identified in women’s adiposity traits and psychiatric disorders. ‘b’ Indicates shared genes identified in men’s adiposity traits and psychiatric disorders. Abbreviations: ADHD, attention deficit hyperactivity disorder; ADP, adiposity trait; AN, anorexia nervosa; ASAT, abdominal subcutaneous adipose tissue; BF%, body fat percentage; BIP, bipolar disorder; BMI, body mass index; GFAT, gluteofemoral adipose tissue; LFR, legs–leg fat ratio; MDD, major depressive disorder; PSY, psychiatric disorder; SCZ, schizophrenia; TFR, trunk–trunk fat ratio; VAT, visceral adipose tissue; WHR, waist-to-hip ratio.

The joint-tissue transcriptome-wide association study (TWAS) using the joint-tissue imputation (JTI) approach estimated the associations between genetically predicted expression in up to 14,534 genes and each trait of adiposity and psychiatric disorders among 26 selected tissues (Figs. S3–S5 and Tables S9–S11). In summary, 22 specific genes were determined as shared genes associated with adiposity traits and psychiatric disorders, with 18, 8, and 5 shared genes for general, women’s, and men’s adiposity traits, respectively (Fig. 3). For example, the genetically predicted gene expression of *MPHOSPH9* was significantly associated with WHR, LFR, and trunk–trunk fat ratio (TFR) (*Z*-score ranged from −8.53 to 5.14) and ADHD and SCZ (*Z*-score ranged from −4.69 to 6.57) across 16 tissues. The genetically predicted gene expression of *RTN4RL1, DENND1A, SP4, NEGR1, TMEM106B,* and *TSNARE1* manifested consistent tissue-specific association directions with adiposity and psychiatric traits, while opposite directions were observed for *SNX19, FKBP2, HAPLN4, NT5C2, TMEM219,* and *ARFGEF2*.

The gene-based integrative fine-mapping through conditional TWAS (GIFT) approach further filtered 17 causal shared genes across 26 tissues, including 3 genes (i.e. *DENND1B, CTNNB1,* and *ARFGEF2*) for women’s adiposity traits only (Table 1). The genetic expression of *SP4* showed consistent association directions with adiposity traits and psychiatric disorders among all tissues (Fig. S6 and Table S12). Inverse associations of gene expression of *NEGR1* with two groups of traits were observed between adiposity tissue and brain. For example, *BMAL1* was identified as a causal shared gene between adiposity traits (i.e., BMI, waist-to-hip ratio [WHR], and BF%) and AN (Table 1), which was also applied to women’s and men’s adiposity traits (i.e., BMI and WHR) and AN (Figs. S7 and S8, Tables S13 and S14).

**Table 1.** Summary of 17 causal shared genes between adiposity traits and psychiatric disorders.^a^

Symbol	CHR: position	Pairwise traits		Drug-gene interaction information		
		Adiposity traits	Psychiatric disorders	Druggable category	Interaction drug	Indication of interaction drug
***RERE*** ^b^ ^,^ ^c^ ^,^ ^d^	1:8412457–8908980	BF%, WHR	MDD, SCZ	Transcription factor	NA	NA
***NEGR1*** ^c^ ^,^ ^d^	1:71861626–72748222	BMI	MDD	Druggable genome	NA	NA
***DENND1B*** ^d^ ^,^ ^e^	1:197473878–197744826	BMI	MDD	NA	NA	NA
***CTNNB1*** ^c^ ^,^ ^e^	3:41236232–41301587	LFR, TFR	AN	Clinically actionable, drug resistance, druggable genome, transcription factor, transcription factor complex	Cabozantinib S-Malate, Celecoxib, Cetuximab, Cyclophosphamide Anhydrous, Dexamethasone, Everolimus, Fluorescein Sodium, Imatinib, Lenalidomide, Letrozole, Nirogacestat, Osimertinib, Sorafenib, Temsirolimus, Thalidomide, Trametinib Dimethyl Sulfoxide	Anti-inflammatory agent, antineoplastic agent, glucocorticoid, immunosuppressant, non-steroidal anti-inflammatory drug, treatment of Meniere’s disease
***TMEM106B*** ^b^ ^,^ ^c^ ^,^ ^d^	7:12250896–12282993	BMI	MDD	Transporter	NA	NA
***SP4*** ^c^ ^,^ ^d^	7:21467661–21554440	BMI	BIP, SCZ	Transcription factor	NA	NA
***TSNARE1*** ^d^	8:143293441–143484543	BMI	SCZ	NA	NA	NA
***DENND1A*** ^d^	9:126141933–126692431	BMI, WHR	MDD	NA	NA	NA
***CNNM2*** ^b^ ^,^ ^c^ ^,^ ^d^	10:104678051–104849979	BMI, WHR	SCZ	Transporter	Mercaptopurine	Antineoplastic agent
***NT5C2*** ^c^ ^,^ ^d^	10:104846942–105037362	BMI	SCZ	Clinically actionable, drug resistance, enzyme	Cytarabine, Didanosine, Mercaptopurine, Peginterferon Alfa-2a, Thioguanine	Antineoplastic agent, immunomodulatory agent, treatment of hepatitis B and C
***BMAL1*** ^b^ ^,^ ^d^ ^,^ ^f^	11:13298199–13408813	BF%, BMI, WHR	AN	NA	NA	NA
***MPHOSPH9*** ^d^	12:123636867–123728561	LFR, TFR, WHR	ADHD, SCZ	NA	NA	NA
***CCDC92***	12:124403207–124457378	GFAT, WHR	SCZ	NA	NA	NA
***YLPM1*** ^d^	14:75230019–75326138	WHR	MDD	NA	NA	NA
***TAOK2*** ^c^ ^,^ ^d^	16:29985189–30003582	BF%, BMI, WHR, AFR	BIP, SCZ	Druggable genome, enzyme, kinase, serine threonine kinase	NA	NA
***RTN4RL1*** ^c^	17:1837971–1928628	BF%, BMI	BIP	Cell surface, druggable genome	NA	NA
***ARFGEF2*** ^c^ ^,^ ^d^ ^,^ ^e^	20:47538248–47653230	AFR	PTSD	Kinase	NA	NA

a This table shows 17 causal shared genes between adiposity traits and psychiatric disorders. The intersection of shared genes identified by PLACO and COLOC from linkage pairs of adiposity traits and psychiatric disorders were retained for TWAS analysis. Then, the JTI TWAS and GIFT analysis filtered 17 causal shared genes with significant associations between gene expression levels and both groups of traits across 26 tissues.

b Indicates that the causal shared genes identified in the primary analysis (general participants) were also identified for men’s adiposity traits and psychiatric disorders.

c Indicates causal shared genes with the “Druggable category” information. The concept and definition of the “Druggable category” were derived from the Drug Gene Interaction database. Only the indication of approved drugs related to causal genes (i.e., having drug-gene interaction) are shown.

d Indicates the linkage pairs of the causal shared genes have causal associations in the UVMR analysis.

e Indicates causal shared genes identified only in women’s adiposity traits and psychiatric disorders.

f Indicates that the causal shared genes identified in the primary analysis (general participants) were also identified for women’s adiposity traits and psychiatric disorders.

Abbreviations: ADHD, attention deficit hyperactivity disorder; AFR, arms–arm fat ratio; AN, anorexia nervosa; BF%, body fat percentage; BIP, bipolar disorder; BMI, body mass index; GFAT, gluteofemoral adipose tissue; LFR, legs–leg fat ratio; MDD, major depressive disorder; NA, not available; PTSD, post-traumatic stress disorder; SCZ, schizophrenia; TFR, trunk–trunk fat ratio; WHR, waist-to-hip ratio.

### Biological function and druggability information

Gene set enrichment analysis indicated that the potential functions of the 17 causal shared genes were mainly in biological processes and signaling pathways, including neurodevelopment, neuronal function, cellular transport, and developmental biology (Table S15). The Drug Gene Interaction database (DGIdb) revealed four causal genes (*NEGR1, CTNNB1, TAOK2*, and *RTN4RL1*) in the druggable genome and two clinically actionable genes (*CTNNB1* and *NT5C2*; Table 1).

### Bidirectional MR estimates for causal associations

**Figure 4.**
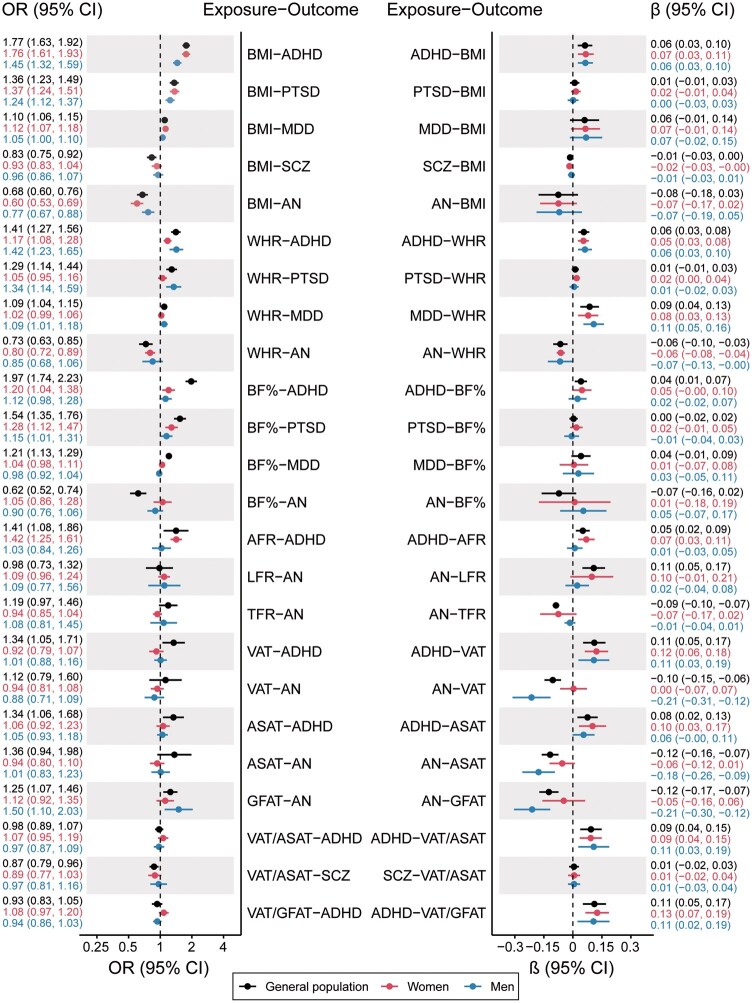
Bidirectional causal associations between adiposity traits and psychiatric disorders identified by UVMR. Causal associations between adiposity traits and psychiatric disorders with FDR-adjusted *P* < 0.05 are shown in this figure (results of all associations see Tables S16 and S17). Spots and error bars indicate the ORs and 95% CIs, respectively, with primary results and the results for women’s and men’s adiposity traits depicted in black, red, and blue. Abbreviations: ADHD, attention deficit hyperactivity disorder; AFR, arms–arm fat ratio; AN, anorexia nervosa; ANX, anxiety; ASAT, abdominal subcutaneous adipose tissue; BF%, body fat percentage; BIP, bipolar disorder; BMI, body mass index; CI, confidence interval; FDR, false discovery rate; GFAT, gluteofemoral adipose tissue; LFR, legs–leg fat ratio; MDD, major depressive disorder; OR, odds ratio; PF%, pancreas fat percentage; PTSD, post-traumatic stress disorder; SCZ, schizophrenia; TFR, trunk–trunk fat ratio; UVMR, univariable MR; VAT, visceral adipose tissue; WHR, waist-to-hip ratio.

Univariable two-sample MR (UVMR) analysis identified five bidirectional associations between the two groups of traits, including positive bidirectional associations of BMI with ADHD and of WHR with ADHD and MDD, an inverse bidirectional association between WHR and AN, and a positive association between gluteofemoral adipose tissue (GFAT) and AN but an inverse association between AN and GFAT (Fig. 4, Tables S16 and S17). These results were highly consistent with using women’s and men’s adiposity data (Fig. 4). Nineteen unidirectional causal associations between adiposity traits and psychiatric disorders were identified (Fig. 4, Tables S16 and S17).

These inverse-variance weighted (IVW) results were supported by multiple sensitivity analyses (Tables S16–S19). The *F*-statistics ranged from 22.86 to 166.48, suggesting that the MR results were less likely to be biased by weak instruments. MRlap results indicated that the partial overlap in samples between exposure and outcome datasets might not bias the causal estimations (Tables S20 and S21).

## Discussion

**Figure 5.**
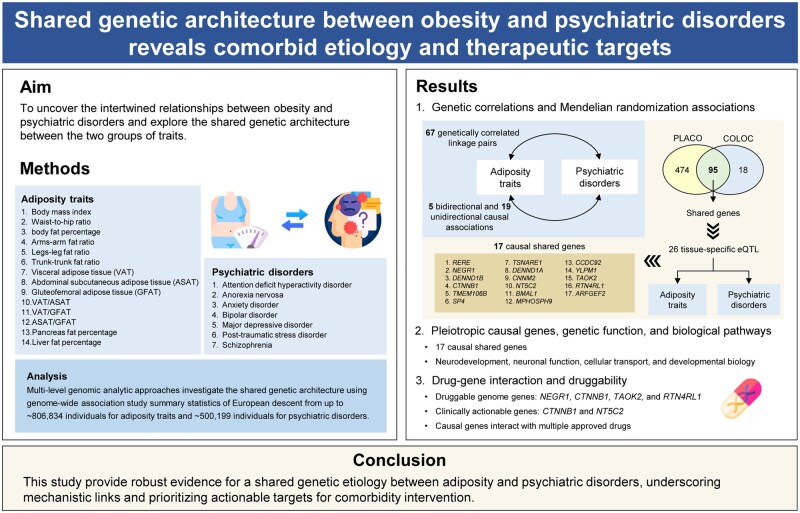
Graphical summary of this study. Abbreviations: COLOC, Bayesian colocalization test; eQTL, expression quantitative trait locus; PLACO, pleiotropic analysis under composite null hypothesis. The elements in the graphic summary were designed using resources from Flaticon.com.

This study dissected shared genetic architecture in the etiology of adiposity and psychiatric disorders from the perspectives of genetic correlations, shared genetic factors, and causal relationships in a multi-level genetic analytic framework (Fig. 5). Based on the 67 genetically correlated linkage pairs established by the LDSC and HDL analyses between 14 adiposity traits and 7 psychiatric disorders, the PLACO and COLOC analyses identified 95 candidate shared genes between the two groups of traits. The JTI TWAS analysis then filtered 22 shared genes (with 18, 8, and 5 genes for general, women’s, and men’s adiposity traits, respectively) by estimating the associations of genetically predicted expression with adiposity traits and psychiatric disorders in 26 tissues. Furthermore, the GIFT approach retained 17 causal shared genes, including 3 genes only for women’s adiposity traits. The gene set enrichment analysis and the DGIdb indicated that the 17 causal shared genes were mainly involved in neurodevelopment, neuronal function, cellular transport, and developmental biology, wherein *NEGR1, CTNNB1, TAOK2,* and *RTN4RL1* were located in the druggable genome, and *CTNNB1* and *NT5C2* were clinically actionable genes. Finally, the MR analysis supported 5 bidirectional and 19 unidirectional causal associations between 11 adiposity traits (excluding abdominal subcutaneous adipose tissue [ASAT]/GFAT, liver fat percentage [LF%], and pancreas fat percentage [PF%]) and 5 psychiatric disorders (ADHD, AN, PTSD, MDD, and SCZ). These findings provided confirmatory evidence for bidirectional and interrelated relationships between adiposity and psychiatric disorders, identified shared multi-level genetic underpinnings, and revealed exploratory evidence of pleiotropic druggable genes, paving the way for integrating shared genetic targets and developing holistic approaches to prevent and manage both groups of conditions.

Our investigation yielded three main findings. First, we quantified broader genetic correlations and MR associations between comprehensive adiposity traits (covering conventional adiposity traits, accurately measured body fat distributions, different sites of body fat ratios, and sex-specific adiposity traits) and representative psychiatric disorders, extending previous evidence from observational and MR studies that associated obesity and higher BMI with elevated risks of depression, ADHD, or PTSD across populations [3, 4, 17]. Interestingly, our MR results identified positive causal effects of adiposity traits (i.e. BMI, WHR, BF%, arms–arm fat ratio [AFR], visceral adipose tissue [VAT], and ASAT) on ADHD, PTSD, and MDD, and vice versa, implying a vicious circle between adiposity and these psychiatric disorders. Immuno-inflammatory, hypothalamic–pituitary-adrenal (HPA) axis dysregulation, gut dysbiosis, insulin resistance, stress, and unhealthy dietary habits might be involved in the mutually fostering relationships between adiposity and these psychiatric disorders [6, 7, 18, 19]. On the other hand, we observed inverse effects of adiposity traits (i.e. BMI, WHR, and BF%) on SCZ and AN, as well as an inverse effect of AN on adiposity (i.e. WHR, TFR, VAT, ASAT, and GFAT). One recent MR study using multi-ancestry GWAS summary statistics of European and East Asian populations reported a null significant effect of BMI on SCZ [5], inconsistent with our finding using GWAS data from European populations, implicating potential heterogeneity in genetic background between BMI and SCZ, with future genetic and mechanistic investigations needed to clarify this complicated relationship. In contrast, the impaired homeostatic control of food intake and energy expenditure, the disturbed hormonal (e.g. the anorexigenic hormone leptin and the orexigenic hormone ghrelin) signaling, and the pathological changes in hypothalamic functioning may offer more harmonious explanations for the observed reverse associations between AN and lower body fat accumulations [20]. These bidirectional associations from MR analysis suggested the genetically predicted associations and potential shared genetic architecture between adiposity traits and psychiatric disorders, rather than proving the clinically definitive causal pathways or bidirectional feedback between the two groups of traits. These results provide biological insights for researchers and clinicians to enable more precise prevention for individuals with a genetic predisposition to obesity and psychiatric disorders in the future.

Second, we delineated an intrinsic genetic underpinning between adiposity and psychiatric disorders, providing nuanced evidence on pleiotropic variants and genes, genetic expressions across tissues, causal features of genes, and genetic function and biological pathways. Several shared biological mechanisms, including pro-inflammatory cytokines, stress, and nervous and endocrine system dysregulation [6, 7], as well as shared risk factors, including lower educational attainment and unhealthy lifestyle behaviors [21], may directly or indirectly affect the intertwined relationships between adiposity and psychiatric disorders [7]. Intriguingly, growing evidence hints at the syngeneic functions for the identified shared genes we found in adiposity traits and psychiatric disorders. For instance, the obesity susceptibility gene *NEGR1* [22] is related to the protein and transcript level changes of neurodevelopment and psychiatric disorders [23, 24]. The circadian clock gene *BMAL1* plays an essential role in muscle function and lipid metabolism [25, 26] and has also been implicated in neuropsychiatric disorders [27]. Previous experimental and genetic studies supported that *RERE, CTNNB1, SP4, TSNARE1, CNNM2, NT5C2, RTN4RL1, ARFGEF2, TAOK2, TMEM106B,* and *DENND1B* are involved in multiple neuropsychiatric disorders, neurodevelopment, or brain aging [28–36]. Among these genes, *DENND1B, TAOK2,* and *TMEM106B* have also been identified as the human obesity gene or playing key roles in inducing obesity in animal models [37–39]. In addition, our gene set enrichment analysis results supported that the identified shared genes are involved in potential biological mechanisms of neural development, signaling pathways on obesity, or immune responses, such as dendrite development (e.g. *RERE, TAOK2,* and *TMEM106B*), negative regulation of the canonical Wnt signaling pathway (e.g. *CTNNB1*), or positive regulation of T cell cytokine production (e.g. *DENND1B*). The causal shared genes partially explain the findings from the MR analysis, providing evidence for assessing comorbidity risk in genetically susceptible individuals and for investigating the underlying mechanisms.

Third, of identified causal shared genes, we outlined four genes (i.e. *NEGR1, CTNNB1, TAOK2,* and *RTN4RL1*) as druggable genome, which may have interactions with drugs or potential therapeutic benefits [40], and six shared genes (i.e. *NT5C2, ARFGEF2, RERE, SP4, CNNM2,* and *TMEM106B*) in other druggable categories, including clinically actionable, drug resistance, enzyme, kinase, transcription factor, and transporter. Moreover, the drug–gene interaction information suggested that three shared genes, including *CTNNB1, CNNM2,* and *NT5C2*, had interactions with multiple approved drugs (e.g. anti-inflammatory agent, antineoplastic agent, glucocorticoid, immunosuppressant, and non-steroidal anti-inflammatory drug; Table 1). These pleiotropic druggable genes hold feasibility in cautioning dual-action medication, enhancing medication efficacy, and promoting personalized therapeutic techniques evolving to confront the clinical and public health challenges from obesity and psychiatric disorders.

In summary, this study enlightened the shared genetic architecture and the bidirectional relationships between adiposity traits and psychiatric disorders from the perspectives of genetic correlation and causality, as well as gene expression, function, and druggability. Our findings illuminate the underpinning genetic etiology of obesity and psychiatric disorders, enlightening avenues for integrating genetic targets and formulating holistic approaches for the management and control of the combined challenge of adiposity and psychiatric disorders.

## Research limitations

The main strengths of this study included the largest GWAS summary statistics to date on body fat distribution and organ fat percentage based on precision measurement techniques, the application of multiple advanced genomic analytic approaches, and the strict research framework to enable reliable and stable results. This work also had several limitations. First, all genetic analyses should fulfill specific key assumptions. Although we used comprehensive sensitivity analyses and multiple methods to ensure the robustness of the results, residual biases caused by the violation of untestable assumptions may exist. Second, although we have identified shared genes underlying the etiology of adiposity and psychiatric disorders, the precise mechanisms of most genes are unknown. Further experimental investigations are warranted to clarify the regulatory function of these genes across different tissues and to validate the co-regulated genes. Third, the findings of this study were derived from the GWAS summary statistics of populations of European descent from over 25 countries in Europe, North America, and Oceania. Therefore, the generalization of our conclusion to other descent populations should be taken with caution.

## Methods

### Research ethics

The ethics review for this study is not required since de-identified and publicly available summary-level GWAS data were utilized in this analysis. Information regarding the ethical approval of the GWAS can be found in the relevant publications referenced within the manuscript.

### Data source

#### Adiposity traits

This study applied GWAS summary statistics of 14 adiposity traits from European descent participants, including BMI, WHR, BF%, AFR, LFR, TFR, VAT volumes, ASAT volumes, GFAT volumes, VAT/ASAT, VAT/GFAT, ASAT/GFAT, LF%, and PF%. Summary statistics for BMI and WHR, which were measured or self-reported, were derived from a meta-analysis of GWAS in up to 806,834 participants from the UK Biobank (UKB) and the Genetic Investigation of ANthropometric Traits Consortium [9]. Summary statistics for BF%, which was measured by body composition analyzer (UKB data field 23099), were from a GWAS in 454,633 participants from the UKB [10]. Summary statistics for AFR, LFR, and TFR, which were measured by body composition analyzer, were from GWASs of 362,499 participants from the UKB [11]. Summary statistics for body fat distribution traits including VAT volumes, ASAT volumes, and GFAT volumes, as well as body fat distribution ratios including VAT/ASAT, VAT/GFAT, and ASAT/GFAT were from GWASs of 39,076 participants (87% European descent) from the UKB [12], where these traits were generated based on body images of MRI. Summary statistics for LF% and PF%, measured by MRI, were derived from GWASs in 32,858 and 25,617 participants from the UKB, respectively [13].

We used GWAS summary statistics for 14 adiposity traits from general populations in the primary analysis. In addition, we also used the sex-specific GWAS summary statistics (available for 12 out of the 14 adiposity traits, except for LF% and PF%) to complement the findings for sex-specific adiposity traits.

#### Psychiatric disorders

GWAS summary statistics of seven psychiatric disorders (ADHD, AN, ANX, bipolar disorder [BIP], MDD, PTSD, and SCZ) were obtained from participants of European descent. GWAS data for ADHD were obtained from a meta-analysis of the Integrative Psychiatric Research Consortium, deCODE, and Psychiatric Genomics Consortium (PGC), including 38,691 cases and 186,843 controls [41]. GWAS data for AN were from 33 datasets with 16,992 cases and 55,525 controls [42]. GWAS data for ANX were from the Anxiety NeuroGenetics STudy Consortium, including 7016 cases and 14,745 controls [43]. GWAS data for BIP were from a meta-analysis of 57 European cohorts totaling 41,917 cases and 371,549 controls [44]. GWAS data for MDD were from the combination of the PGC and UKB, including up to 170,756 cases and 329,443 controls [45]. GWAS data for PTSD and SCZ were from the PGC with 174,659 (23,212 cases and 151,447 controls) and 130,644 participants (53,386 cases and 77,258 controls), respectively [46, 47]. Cases for each psychiatric disorder were defined according to the specific Diagnostic and Statistical Manual of Mental Disorders criteria, International Classification of Diseases codes, or self-reported. Detailed information on each trait is summarized in Table S1.

### Statistical analysis

#### Genetic correlation and shared gene selection

We used the LDSC and HDL approaches to estimate the genome-wide genetic correlations between each adiposity trait and psychiatric disorder (Fig. 1A) [48, 49]. To ensure adequate statistical power for subsequent exploratory analyses of shared variants, a false discovery rate (FDR)-adjusted *P *< 0.05 was considered statistically significant after the multiple testing correction, with 98 tests conducted for correlations between 14 adiposity traits and 7 psychiatric disorders and 84 tests conducted for correlations between 12 sex-specific adiposity traits and 7 psychiatric disorders. Trait pairs exhibiting a statistically significant genetic correlation in at least one of the two methods (LDSC and HDL) were identified as linkage pairs. Among these linkage pairs, we used PLACO to identify the shared variants [50]. Genetic variants with a genome-wide significance threshold of *P_PLACO_* < 5 × 10^−8^ were defined for shared variants. Concurrently, a COLOC analysis was performed to identify the causal shared variants, and the one with the highest posterior probability of hypothesis 4 (PP.H4) was selected in the region of summary PP.H4 > 0.8, between linkage traits [51]. The SNPnexus was applied to annotate the variants based on human reference assembly GRCh37 [52]. To ensure the accuracy and robustness of the identified shared genes through verification by multiple methods, we independently employed PLACO and COLOC analysis for identifying shared variants. After that, under the triangulation framework, an intersection of shared genes identified by both methods was retained for subsequent analysis.

#### Joint-tissue TWAS analysis

We employed the TWAS analysis using the JTI approach to estimate the associations between tissue-specific genetically predicted gene expression levels and traits (Fig. 1B) [53]. A *Z*-score was derived from the estimation of TWAS analysis divided by SE. To deepen our understanding of the heterogeneity of gene function and the potential regulatory mechanisms underlying these comorbidities, we included transcriptomic data from 26 tissues across multiple physiological systems based on their complex relevance to both adiposity and psychiatric disorders for transcriptomic analysis. In addition to two adipose tissues (subcutaneous and visceral), 13 brain regions (amygdala, anterior cingulate cortex, caudate, hippocampus, nucleus accumbens, putamen, substantia nigra, hypothalamus, spinal cord, cerebellar hemisphere, cerebellum, cortex, and frontal cortex), and whole blood, we also incorporated (i) six digestive system tissues (two colon tissues, intestine, stomach, liver, and pancreas) involved in fat–brain regulation through the gut–liver–brain axis, (ii) three endocrine tissues (pituitary gland, adrenal gland, and thyroid gland) associated with the HPA axis, and (iii) skeletal muscle, which participates in bidirectional muscle–brain crosstalk. The multiple testing correction of JTI analysis included tests ranging from 6170 (gene expression with ADHD in brain amygdala) to 14,534 (gene expression with BF% in thyroid) for each adiposity trait and psychiatric disorder within each tissue. Within each tissue type, to minimize false positive findings from multiple testing of numerous candidate genes, we considered only those genes meeting a Bonferroni-adjusted *P *< 0.05 as statistically significant for each analyzed trait.

#### Conditional TWAS analysis

We utilized the two-stage version of the GIFT approach to identify causal shared genes among the genes identified by the JTI (Fig. 1C) [54]. Similar to the JTI TWAS analysis, all genes in the independent linkage disequilibrium (LD) blocks of each shared gene were tested for each trait in the 26 tissues. Genes with causal associations between genetically predicted expression levels and both adiposity traits and psychiatric disorders were retained as the final causal shared genes, with Bonferroni-adjusted *P *< 0.05 controlling multiple testing corrections of GIFT analysis.

#### Gene set enrichment and druggable analysis

We conducted the Gene Ontology [55] and Kyoto Encyclopedia of Genes and Genomes [56] gene set enrichment analysis to investigate the function and pathway of the identified causal shared genes. We then used the DGIdb (dgidb.org) to identify the drug-gene interaction and druggability information of causal shared genes [40].

#### Bidirectional MR analysis

We conducted the bidirectional UVMR analysis to test the causal associations between adiposity traits and psychiatric disorders (Fig. 1D) [10], with MR estimates presented as odds ratios and 95% confidence intervals (CIs) for binary outcomes and β coefficients and 95% CIs for continuous outcomes. Following the LD clumping, data harmonization, and proxy selection, the main results were tested using instrumental variants for adiposity traits and psychiatric disorders, respectively, using the IVW method. After multiple testing corrections, including 98 tests for associations between 14 adiposity traits and 7 psychiatric disorders and 84 tests for 12 sex-specific adiposity traits and 7 psychiatric disorders, an FDR-adjusted *P *< 0.05 was considered statistically significant. To estimate the causal effects when not all the genetic variants were valid, we also employed sensitivity MR analysis, namely weighted median, weighted mode, MR-Egger, and MR pleiotropy residual sum and outlier [57–60]. We then used the MRlap method to validate the MR analysis results for the traits with sample overlap [61].

Genetic correlation analysis was conducted using LDSC software (version 1.0.1). All other analyses were performed using R packages *HDL* (version 1.4.0), *PLACO* (version 0.1.1), *COLOC* (version 5.2.2), *JTI* (version 1.0), *GIFT* (version 1.0), *clusterprofiler* (version 4.8.3), *TwoSampleMR* (version 0.5.6), *MRPRESSO* (version 1.0), and *MRlap* (version 0.0.3.2) in R software (version 4.3.1). A detailed method is presented in Supplementary Methods.

## Supplementary Material

lnag015_Supplementary_Data

## Data Availability

All GWAS summary data used in this study are publicly available. The GWAS summary data for adiposity traits can be retrieved or requested from the original studies or the UKB (BMI and WHR: doi.org/10.1093/hmg/ddy327; body fat percentage: gwas.mrcieu.ac.uk and www.nealelab.is/uk-biobank; body composition analyzer measured fat ratio traits: doi.org/10.1038/s41467-018–08000-4; MRI-derived body fat distribution traits: doi.org/10.1038/s41467-022–30931-2; and organ fat traits: doi.org/10.7554/eLife.65554). The GWAS summary data for psychiatric disorders can be downloaded from the Psychiatric Genomics Consortium (pgc.unc.edu/for-researchers/download-results). All data and results generated in this study could be obtained from the Supplementary Tables.
