# Supplementary material for: Shared genetic architecture between obesity and psychiatric disorders reveals comorbid etiology and therapeutic targets
Source: Life Med. 2026 Apr 22;5(3):lnag015. doi: 10.1093/lifemedi/lnag015 (PMC13249615; doi:10.1093/lifemedi/lnag015)
Supplement: lnag015_Supplementary_Data [file lnag015_supplementary_data.pdf]

Shared genetic architecture between obesity and psychiatric disorders reveals comorbid etiology and therapeutic targets

Dong Liu[1,2,#], Chun Dou[1,2,#], Chaojie Ye[1,2], Mingling Chen[1,2], Lijie Kong[1,2], Zheng Zhu[1,2], Jie Zheng[1,2], Min Xu[1,2], Yu Xu[1,2], Mian Li[1,2], Zhiyun Zhao[1,2], Jieli Lu[1,2], Yuhong Chen[1,2], Zhongshang Yuan[3], Guang Ning[1,2], Weiqing Wang[1,2], Yufang Bi[1,2,*], Tiange Wang[1,2,*]

[1]Department of Endocrine and Metabolic Diseases, Shanghai Institute of Endocrine and Metabolic Diseases, Ruijin Hospital, Shanghai Jiao Tong University School of Medicine, Shanghai 200025, China

[2]Shanghai National Clinical Research Center for Metabolic Diseases, Key Laboratory for Endocrine and Metabolic Diseases of the National Health Commission of the PR China, Shanghai Key Laboratory for Endocrine Tumor, Ruijin Hospital, Shanghai Jiao Tong University School of Medicine, Shanghai 200025, China

[3]Department of Biostatistics, School of Public Health, Cheeloo College of Medicine, Institute for Medical Dataology, Shandong University, Jinan, Shandong 250012, China

[#]These authors contributed equally to this work.

[*]Correspondence: tiange.wang@shsmu.edu.cn (T.W.), byf10784@rjh.com.cn (Y.B.)

Supplementary Materials

Table of contents

Supplementary Methods

References

Supplementary Figure 1. Linkage pairs between women's and men's adiposity traits and psychiatric disorders

Supplementary Figure 2. Shared genes between women's and men's adiposity traits and psychiatric disorders among linkage pairs

Supplementary Figure 3. Associations between genetically predicted expression of shared genes and adiposity traits and psychiatric disorders of general population in 26 tissues using JTI analysis

Supplementary Figure 4. Associations between genetically predicted expression of shared genes and women's adiposity traits and psychiatric disorders in 26 tissues using JTI analysis

Supplementary Figure 5. Associations between genetically predicted expression of shared genes and men's adiposity traits and psychiatric disorders in 26 tissues using JTI analysis

Supplementary Figure 6. Associations between genetically predicted expression of shared genes and adiposity traits and psychiatric disorders of general population in 26 tissues using GIFT analysis

Supplementary Figure 7. Associations between genetically predicted expression of shared genes and women's adiposity traits and psychiatric disorders in 26 tissues using GIFT analysis

Supplementary Figure 8. Associations between genetically predicted expression of shared genes and men's adiposity traits and psychiatric disorders in 26 tissues using GIFT analysis

Supplementary Methods

Pleiotropic analysis under composite null hypothesis (PLACO) and Bayesian colocalization test (COLOC) analysis

The PLACO approach uses Z-scores and P values to detect pleiotropic variants for two binary and/or continuous phenotypes, even if there are overlapping samples [1]. Data harmonization was applied to make sure each variant was in the same direction for both phenotypes. To avoid the false positive results caused by the variants with strong effects in one phenotype but not in another, all the variants with Z-score^2>80 were removed from the analysis.

The advantage of COLOC is using genome-wide association study (GWAS) summary statistics and the Bayes statistical method to estimate the associations for common or rare variants with two phenotypes [2]. Independent genetic variants (r^2<0.001 and window=10,000 kb) that reached the genome-wide significance ($P<5\times10^{-8}$ or $P<5\times10^{-6}$, for the phenotypes that have two or fewer genetic variants) were extracted from the GWAS summary statistics as the leading single nucleotide polymorphism (SNP). All the SNPs located in the ±500 kb window near the leading SNP were extracted from the pair of adiposity traits and psychiatric disorders for COLOC analysis. For the COLOC analysis, five hypotheses were tested, H_0: no association for both phenotypes; H_1: association with phenotype 1 only; H_2: association with phenotype 2 only; H_3: two independent SNPs associated with two phenotypes, respectively; H_4: one SNP associated with both phenotypes. The summary posterior probability of hypothesis 4 (PP.H4)>0.8 indicated that the region contains the shared causal variants for both phenotypes. Finally, the SNP with a highest PP.H4 was selected as the top significant variant.

Joint-tissue imputation (JTI) approach

To conduct the JTI transcriptome-wide association study (TWAS) analysis, we download the Genotype-Tissue Expression (GTEx) pre-trained prediction models from Zenodo (https://doi.org/10.5281/zenodo.3842289) [3]. Then we tested the associations between predicted gene expression and each trait in 26 tissues. After that, we compared candidate-shared genes (i.e., shared genes identified by PLACO and COLOC in the previous step; **Fig. 1A**) in each linkage pair to generate the final shared genes which are associated with both adiposity traits and psychiatric disorders in at least one tissue.

Gene-based integrative fine-mapping through conditional TWAS (GIFT) approach

We followed the two-stage version of GIFT using the GWAS summary statistics and pre-trained weights as input [4]. The detailed manual and example codes can be found on the github (yuanzhongshang.github.io/GIFT). We used the GTEx v8 multi-tissue expression weights from the FUSION approach [5]. Shared genes followed the similar criteria in the JTI analysis were finally retained as the causal shared genes.

Univariable two-sample Mendelian randomization (MR) analysis

The instrumental variants (IVs) used in all MR analyses satisfied three core assumptions: (1) the IVs must be vigorously associated with the exposure in univariable two-sample MR; (2) the IVs must not be associated with confounders of the associations between instruments of each exposure and each outcome; and (3) the effect of IVs on the outcome must go through the exposure. For the phenotypes that have two or fewer candidate IVs such as legs-leg fat ratio and trunk-trunk fat ratio in men, visceral adipose tissue and abdominal subcutaneous adipose tissue in men and women, and post-traumatic stress disorder, we used $P<5\times10^{-6}$ as the threshold. We first utilized the clump_data function ($r^2<0.001$ and window=10,000 kb) in the TwoSampleMR R package to identify the representative genetic variants. Based on the European 1000 genomes reference panel, bi-allelic genetic variants with minor allele frequency>0.01 would be retained for the following process. Each genetic variant was searched in the adiposity traits and psychiatric disorders GWAS summary statistics for the SNP-outcome associations. If a variant was absent from the outcome summary statistics, a high linkage disequilibrium (LD) proxy SNP (European descent, $r^2>0.8$, and window=500 kb) would be searched via the LDproxy tool (https://ldlink.nci.nih.gov/?tab=ldproxy) and replaced the original SNP for the outcome association. SNPs that were absent from the outcome GWAS summary statistics and did not have proxies were discarded from the MR analysis. After the multiple testing correction, an FDR-adjusted $P<0.05$ was considered statistically significant.

The main MR results were tested via the inverse-variance weighted (IVW) method. The IVW method could generate precise MR estimations using multiple valid genetic variants from the GWAS data set [6]. A random-effects IVW method was

used when more than three IVs were available in the MR analysis [7]. Otherwise, a fixed-effect IVW method was used if there are only three or fewer IVs. An average F statistic was estimated for each exposure phenotype. Generally, F statistics greater than 10 supported the strong IVs and indicated that the MR estimates are unlikely to be influenced by weak instrument bias [8]. Furthermore, Cochran's Q test was used to assess the heterogeneity of causal effect estimates [9].

In the sensitivity MR analysis, several robust methods were employed, namely weighted median, weighted mode, MR-Egger, and MR pleiotropy residual sum and outlier (MR-PRESSO) [10-13]. The weighted median method could provide a consistent estimation even when the IV set contains less than 50% invalid genetic variants [10]. The weighted mode could provide a consistent causal estimate regardless of horizontal pleiotropy in the majority of IVs when the ZEro Modal Pleiotropy Assumption is satisfied [11]. The MR-Egger regression could yield a consistent result even if all genetic variants are invalid under the Instrument Strength Independent of Direct Effect assumption [12]. The MR-PRESSO was used to obtain results when removing heterogenous IVs [13].

References

1. D. Ray, N. Chatterjee, A powerful method for pleiotropic analysis under composite null hypothesis identifies novel shared loci between Type 2 Diabetes and Prostate Cancer. *PLoS Genet* **16**, e1009218 (2020).
2. C. Giambartolomei, D. Vukcevic, E. E. Schadt, L. Franke, A. D. Hingorani, C. Wallace, V. Plagnol, Bayesian Test for Colocalisation between Pairs of Genetic Association Studies Using Summary Statistics. *PLoS Genet* **10**, e1004383 (2014).
3. D. Zhou, Y. Jiang, X. Zhong, N. J. Cox, C. Liu, E. R. Gamazon, A unified framework for joint-tissue transcriptome-wide association and Mendelian randomization analysis. *Nat Genet* **52**, 1239-1246 (2020).
4. L. Liu, R. Yan, P. Guo, J. Ji, W. Gong, F. Xue, Z. Yuan, X. Zhou, Conditional transcriptome-wide association study for fine-mapping candidate causal genes. *Nat Genet* **56**, 348-356 (2024).
5. A. Gusev, A. Ko, H. Shi, G. Bhatia, W. Chung, B. W. J. H. Penninx, R. Jansen, E. J. C. de Geus, D. I. Boomsma, F. A. Wright, P. F. Sullivan, E. Nikkola, M. Alvarez, M. Civelek, A. J. Lusis, T. Lehtimäki, E. Raitoharju, M. Kähönen, I. Seppälä, O. T. Raitakari, J. Kuusisto, M. Laakso, A. L. Price, P. Pajukanta, B. Pasaniuc, Integrative approaches for large-scale transcriptome-wide association studies. *Nat Genet* **48**, 245-252 (2016).
6. S. Burgess, A. Butterworth, S. G. Thompson, Mendelian Randomization Analysis With Multiple Genetic Variants Using Summarized Data. *Genet Epidemiol* **37**, 658-665 (2013).
7. O. O. Yavorska, S. Burgess, MendelianRandomization: an R package for performing Mendelian randomization analyses using summarized data. *Int J Epidemiol* **46**, 1734-1739 (2017).
8. S. Burgess, S. G. Thompson, C. C. G. Collaboration, Avoiding bias from weak instruments in Mendelian randomization studies. *Int J Epidemiol* **40**, 755-764 (2011).
9. F. D. Greco M, C. Minelli, N. A. Sheehan, J. R. Thompson, Detecting pleiotropy in Mendelian randomisation studies with summary data and a continuous outcome. *Stat Med* **34**, 2926-2940 (2015).

10. J. Bowden, G. Davey Smith, P. C. Haycock, S. Burgess, Consistent Estimation in Mendelian Randomization with Some Invalid Instruments Using a Weighted Median Estimator. *Genet Epidemiol* **40**, 304-314 (2016).
11. F. P. Hartwig, G. Davey Smith, J. Bowden, Robust inference in summary data Mendelian randomization via the zero modal pleiotropy assumption. *Int J Epidemiol* **46**, 1985-1998 (2017).
12. J. Bowden, G. Davey Smith, S. Burgess, Mendelian randomization with invalid instruments: effect estimation and bias detection through Egger regression. *Int J Epidemiol* **44**, 512-525 (2015).
13. M. Verbanck, C. Y. Chen, B. Neale, R. Do, Detection of widespread horizontal pleiotropy in causal relationships inferred from Mendelian randomization between complex traits and diseases. *Nat Genet* **50**, 693-698 (2018).

Supplementary Figure 1. Linkage pairs between women's and men's adiposity traits and psychiatric disorders.

A. Pairs of women's adiposity traits and psychiatric disorders with significant genetic correlation in either LDSC or HDL. B. Pairs of men's adiposity traits and psychiatric disorders with significant genetic correlation in either LDSC or HDL.

Abbreviations: ADHD, attention deficit hyperactivity disorder; AFR, arms-arm fat ratio; AN, anorexia nervosa; ANX, anxiety; ASAT, abdominal subcutaneous adipose tissue; BF%, body fat percentage; BIP, bipolar disorder; BMI, body mass index; GFAT, gluteofemoral adipose tissue; HDL, high-definition likelihood; LDSC, linkage disequilibrium score regression; LFR, legs-leg fat ratio; MDD, major depressive disorder; PTSD, post-traumatic stress disorder; SCZ, schizophrenia; TFR, trunk-trunk fat ratio; VAT, visceral adipose tissue; WHR, waist-to-hip ratio.

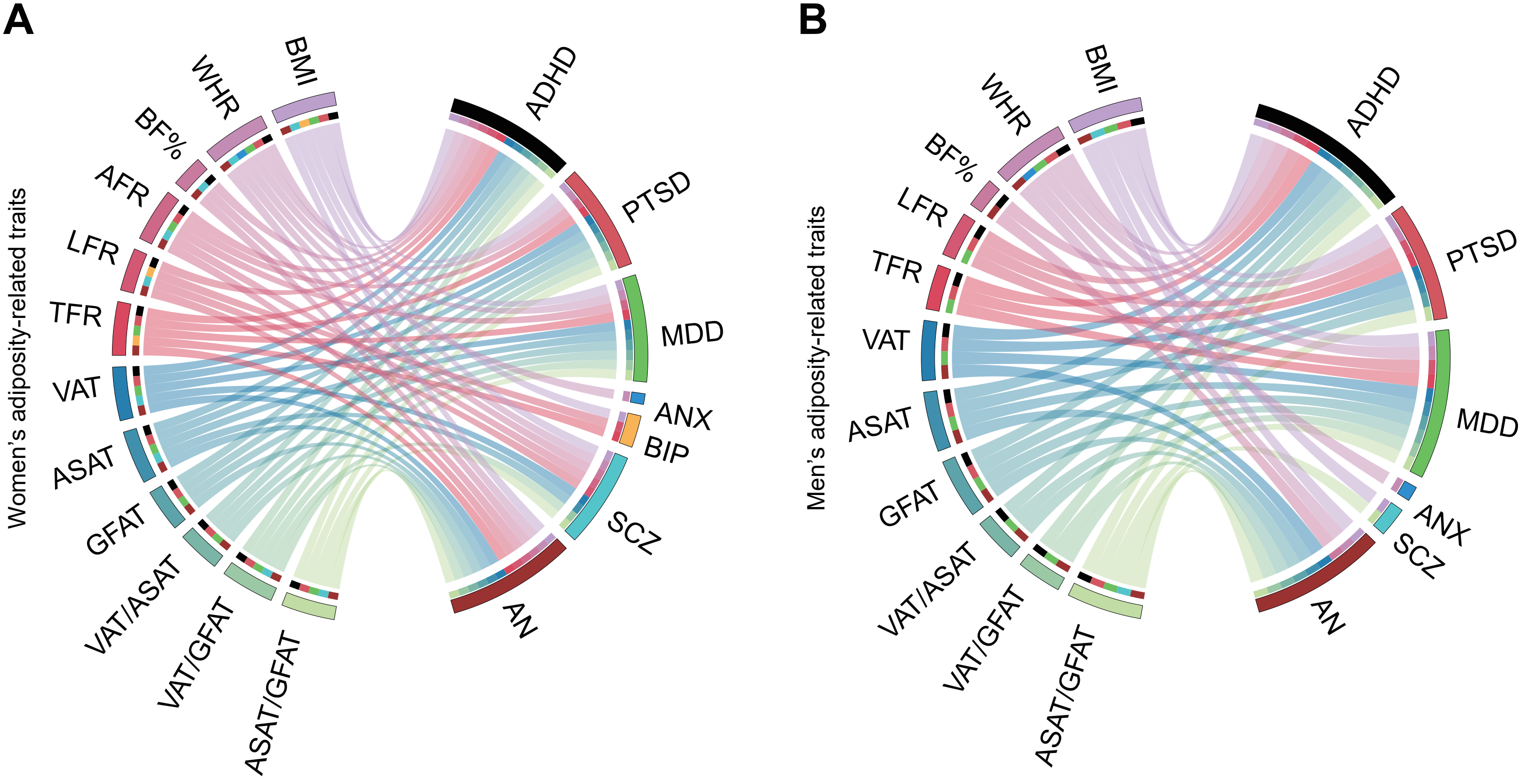

A
Women's adiposity-related traits
BMI
WHR
BF%
AFR
LFR
TFR
VAT
ASAT
GFAT
VAT/ASAT
VAT/GFAT
ASAT/GFAT
ADHD
PTSD
MDD
ANX
BIP
SCZ
AN
B
Men's adiposity-related traits
BMI
WHR
BF%
LFR
TFR
VAT
ASAT
GFAT
VAT/ASAT
VAT/GFAT
ASAT/GFAT
ADHD
PTSD
MDD
ANX
SCZ
AN

Supplementary Figure 2. Shared genes between women's and men's adiposity traits and psychiatric disorders among linkage pairs.

A. Shared genes for each linkage pair using women's adiposity traits and psychiatric disorders.

B. Shared genes for each linkage pair using men's adiposity traits and psychiatric disorders. The linked points in each column indicate the adiposity traits and psychiatric disorders for each linkage pair. The bar chart indicates the number of shared genes in the intersection of PLACO and COLOC analysis results. The abbreviations of adiposity traits are in red and the abbreviations of psychiatric disorders are in blue.

Abbreviations: ADHD, attention deficit hyperactivity disorder; AFR, arms-arm fat ratio; AN, anorexia nervosa; ANX, anxiety; ASAT, abdominal subcutaneous adipose tissue; BF%, body fat percentage; BIP, bipolar disorder; BMI, body mass index; COLOC, Bayesian colocalization test; GFAT, gluteofemoral adipose tissue; LFR, legs-leg fat ratio; MDD, major depressive disorder; PLACO, pleiotropic analysis under composite null hypothesis; PTSD, post-traumatic stress disorder; SCZ, schizophrenia; TFR, trunk-trunk fat ratio; VAT, visceral adipose tissue; WHR, waist-to-hip ratio.

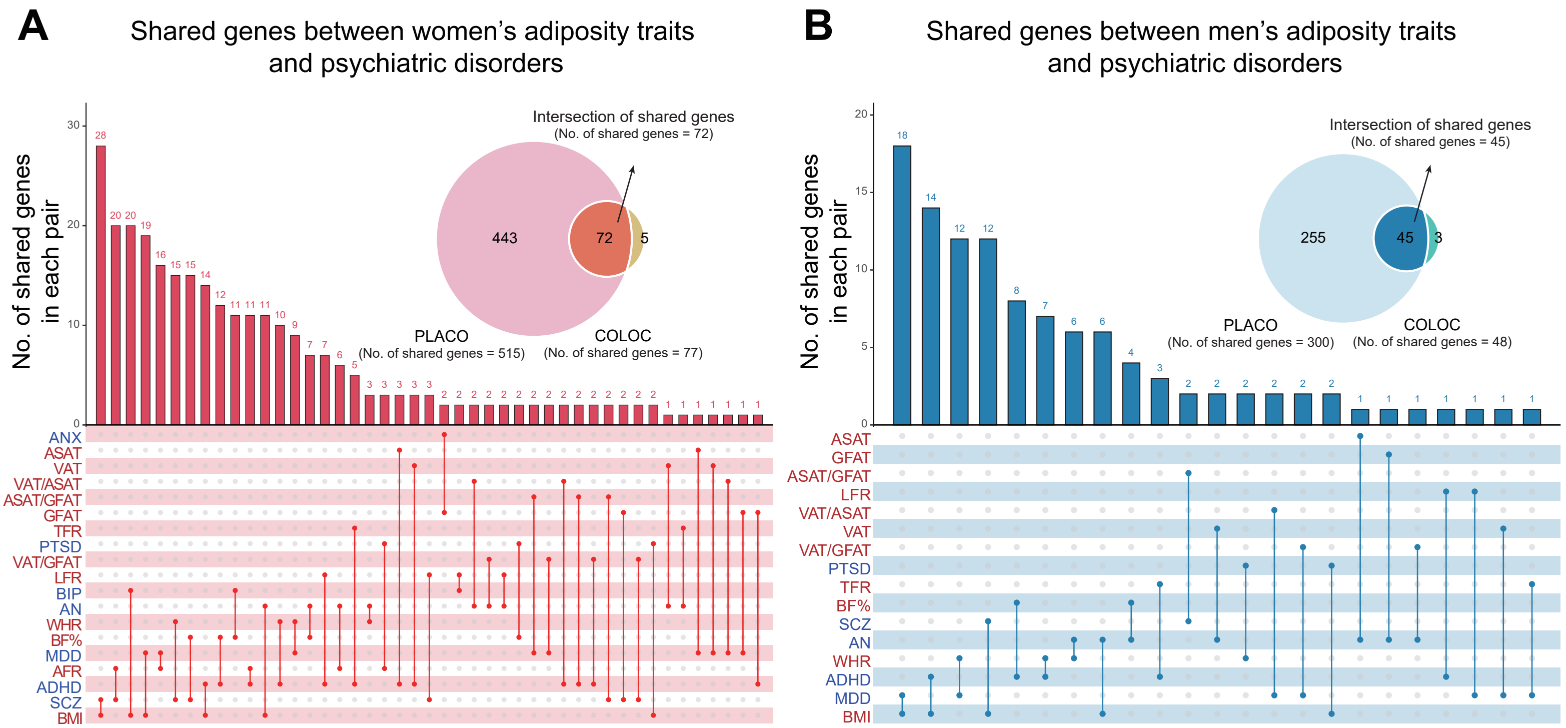
A
Shared genes between women's adiposity traits and psychiatric disorders
No. of shared genes in each pair
Intersection of shared genes
(No. of shared genes = 72)
443
72
5
PLACO
(No. of shared genes = 515)
COLOC
(No. of shared genes = 77)
ANX
ASAT
VAT
VAT/ASAT
ASAT/GFAT
GFAT
TFR
PTSD
VAT/GFAT
LFR
BIP
AN
WHR
BF%
MDD
AFR
ADHD
SCZ
BMI
B
Shared genes between men's adiposity traits and psychiatric disorders
No. of shared genes in each pair
Intersection of shared genes
(No. of shared genes = 45)
255
45
3
PLACO
(No. of shared genes = 300)
COLOC
(No. of shared genes = 48)
ASAT
GFAT
ASAT/GFAT
LFR
VAT/ASAT
VAT
VAT/GFAT
PTSD
TFR
BF%
SCZ
AN
WHR
ADHD
MDD
BMI

Supplementary Figure 3. Associations between genetically predicted expression of shared genes and adiposity traits and psychiatric disorders of general population in 26 tissues using JTI analysis.

Grey blocks indicate the association was unavailable. Blocks with numbers only indicate the unadjusted $P \geq 0.05$. Blocks with numbers and "*" indicate the unadjusted $P < 0.05$. Blocks with numbers and "**" indicate the Bonferroni-adjusted $P < 0.05$.

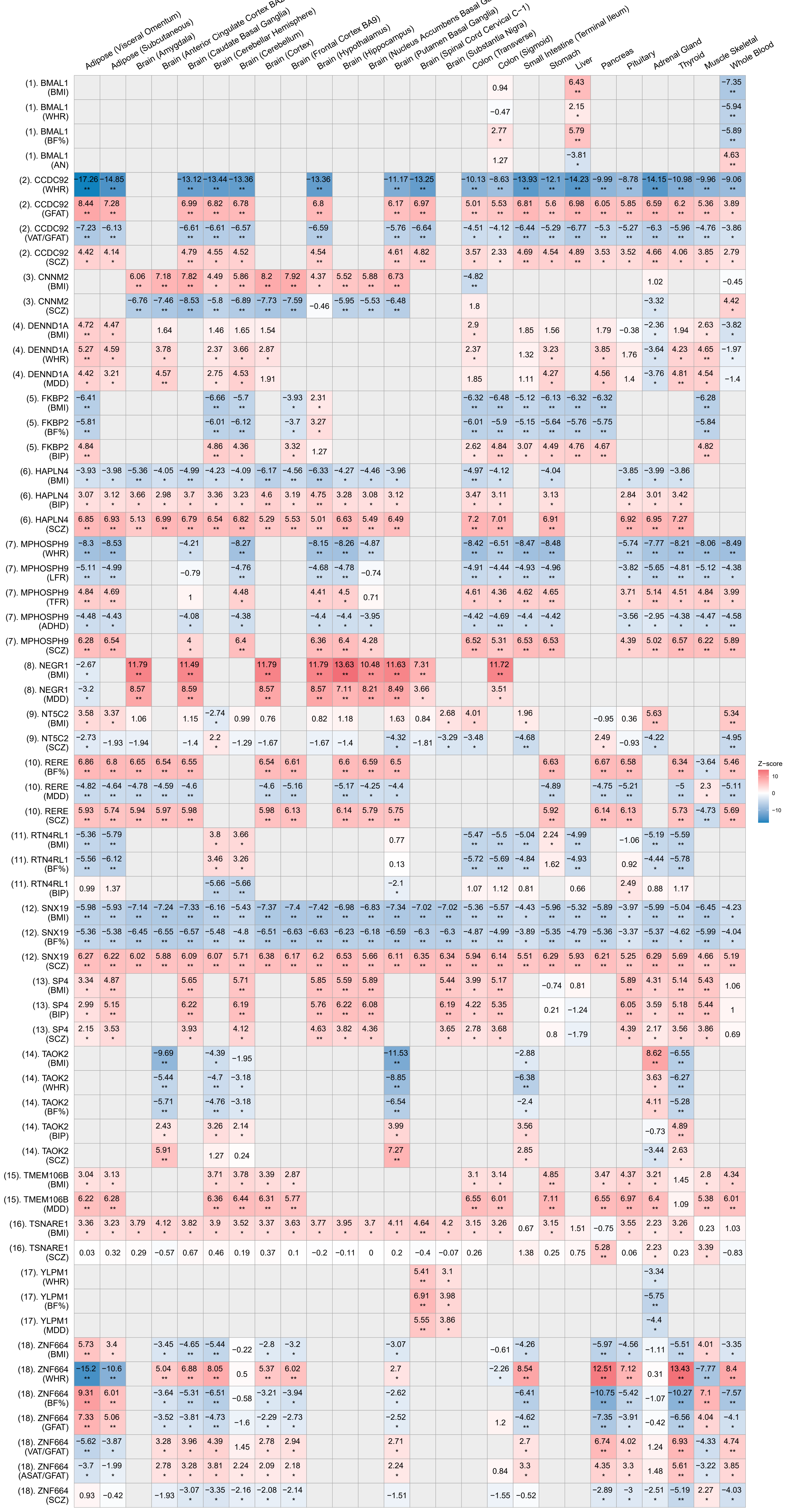
Adipose (Visceral Omentum)
Adipose (Subcutaneous)
Brain (Amygdala)
Brain (Anterior Cingulate Cortex BA24)
Brain (Caudate Basal Ganglia)
Brain (Cerebellar Hemisphere)
Brain (Cerebellum)
Brain (Cortex)
Brain (Frontal Cortex BA9)
Brain (Hypothalamus)
Brain (Hippocampus)
Brain (Nucleus Accumbens Basal Ganglia)
Brain (Putamen Basal Ganglia)
Brain (Spinal Cord Cervical C−1)
Brain (Substantia Nigra)
Colon (Transverse)
Colon (Sigmoid)
Small Intestine (Terminal Ileum)
Stomach
Liver
Pancreas
Pituitary
Adrenal Gland
Thyroid
Muscle Skeletal
Whole Blood
(1). BMAL1 (BMI)
(1). BMAL1 (WHR)
(1). BMAL1 (BF%)
(1). BMAL1 (AN)
(2). CCDC92 (WHR)
(2). CCDC92 (GFAT)
(2). CCDC92 (VAT/GFAT)
(2). CCDC92 (SCZ)
(3). CNNM2 (BMI)
(3). CNNM2 (SCZ)
(4). DENND1A (BMI)
(4). DENND1A (WHR)
(4). DENND1A (MDD)
(5). FKBP2 (BMI)
(5). FKBP2 (BF%)
(5). FKBP2 (BIP)
(6). HAPLN4 (BMI)
(6). HAPLN4 (BIP)
(6). HAPLN4 (SCZ)
(7). MPHOSPH9 (WHR)
(7). MPHOSPH9 (LFR)
(7). MPHOSPH9 (TFR)
(7). MPHOSPH9 (ADHD)
(7). MPHOSPH9 (SCZ)
(8). NEGR1 (BMI)
(8). NEGR1 (MDD)
(9). NT5C2 (BMI)
(9). NT5C2 (SCZ)
(10). RERE (BF%)
(10). RERE (MDD)
(10). RERE (SCZ)
(11). RTN4RL1 (BMI)
(11). RTN4RL1 (BF%)
(11). RTN4RL1 (BIP)
(12). SNX19 (BMI)
(12). SNX19 (BF%)
(12). SNX19 (SCZ)
(13). SP4 (BMI)
(13). SP4 (BIP)
(13). SP4 (SCZ)
(14). TAOK2 (BMI)
(14). TAOK2 (WHR)
(14). TAOK2 (BF%)
(14). TAOK2 (BIP)
(14). TAOK2 (SCZ)
(15). TMEM106B (BMI)
(15). TMEM106B (MDD)
(16). TSNARE1 (BMI)
(16). TSNARE1 (SCZ)
(17). YLPM1 (WHR)
(17). YLPM1 (BF%)
(17). YLPM1 (MDD)
(18). ZNF664 (BMI)
(18). ZNF664 (WHR)
(18). ZNF664 (BF%)
(18). ZNF664 (GFAT)
(18). ZNF664 (VAT/GFAT)
(18). ZNF664 (ASAT/GFAT)
(18). ZNF664 (SCZ)
Z−score
10
0
−10

Supplementary Figure 4. Associations between genetically predicted expression of shared genes and women's adiposity traits and psychiatric disorders in 26 tissues using JTI analysis.

Grey blocks indicate the association was unavailable. Blocks with numbers only indicate the unadjusted $P \geq 0.05$. Blocks with numbers and "*" indicate the unadjusted $P < 0.05$. Blocks with numbers and "**" indicate the Bonferroni-adjusted $P < 0.05$.

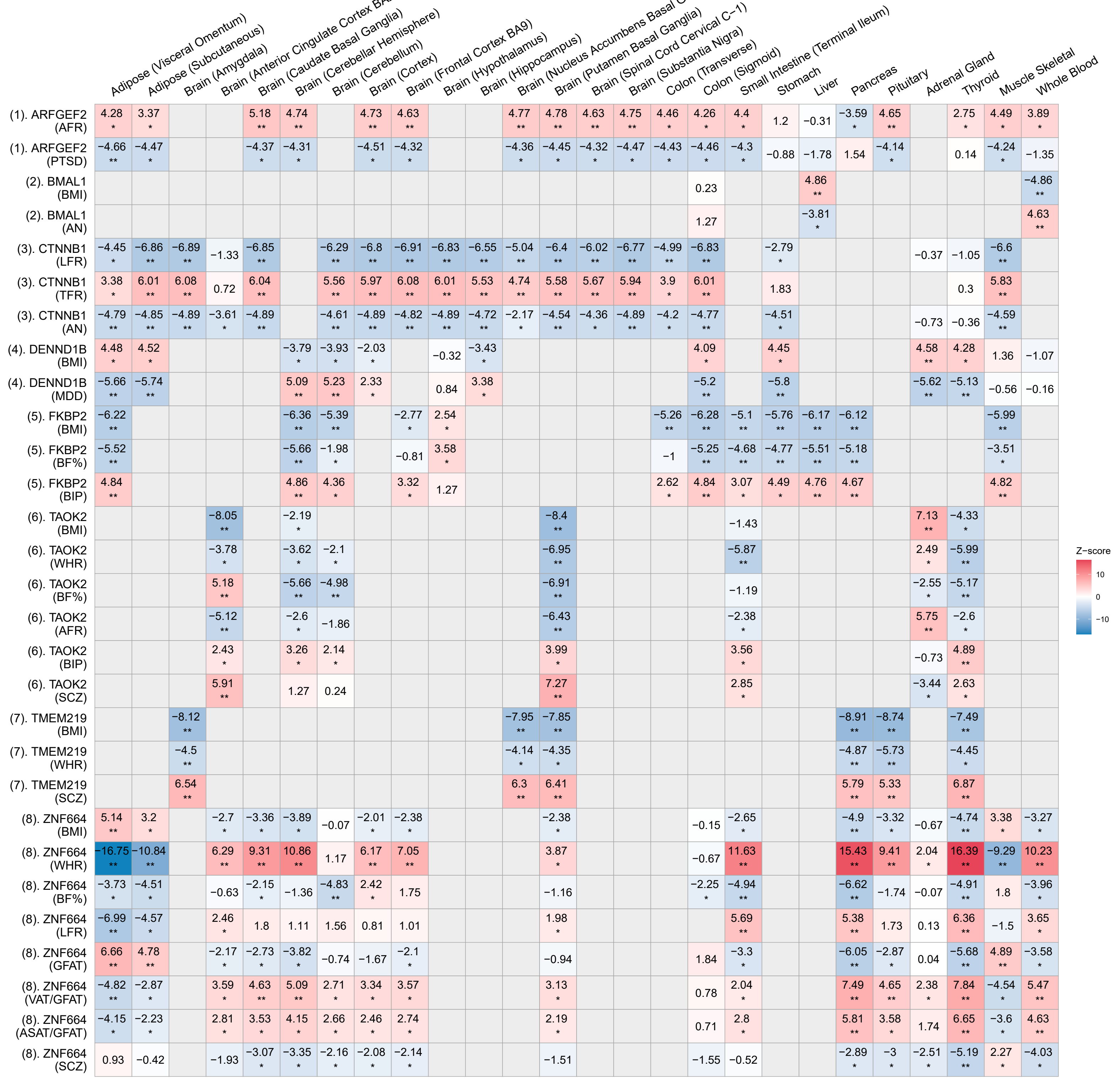

Adipose (Visceral Omentum)
Adipose (Subcutaneous)
Brain (Amygdala)
Brain (Anterior Cingulate Cortex BA24)
Brain (Caudate Basal Ganglia)
Brain (Cerebellar Hemisphere)
Brain (Cerebellum)
Brain (Cortex)
Brain (Frontal Cortex BA9)
Brain (Hypothalamus)
Brain (Hippocampus)
Brain (Nucleus Accumbens Basal Ganglia)
Brain (Putamen Basal Ganglia)
Brain (Spinal Cord Cervical C−1)
Brain (Substantia Nigra)
Colon (Transverse)
Colon (Sigmoid)
Small Intestine (Terminal Ileum)
Stomach
Liver
Pancreas
Pituitary
Adrenal Gland
Thyroid
Muscle Skeletal
Whole Blood
(1). ARFGEF2 (AFR)
(1). ARFGEF2 (PTSD)
(2). BMAL1 (BMI)
(2). BMAL1 (AN)
(3). CTNNB1 (LFR)
(3). CTNNB1 (TFR)
(3). CTNNB1 (AN)
(4). DENND1B (BMI)
(4). DENND1B (MDD)
(5). FKBP2 (BMI)
(5). FKBP2 (BF%)
(5). FKBP2 (BIP)
(6). TAOK2 (BMI)
(6). TAOK2 (WHR)
(6). TAOK2 (BF%)
(6). TAOK2 (AFR)
(6). TAOK2 (BIP)
(6). TAOK2 (SCZ)
(7). TMEM219 (BMI)
(7). TMEM219 (WHR)
(7). TMEM219 (SCZ)
(8). ZNF664 (BMI)
(8). ZNF664 (WHR)
(8). ZNF664 (BF%)
(8). ZNF664 (LFR)
(8). ZNF664 (GFAT)
(8). ZNF664 (VAT/GFAT)
(8). ZNF664 (ASAT/GFAT)
(8). ZNF664 (SCZ)
Z−score
10
0
−10

Supplementary Figure 5. Associations between genetically predicted expression of shared genes and men's adiposity traits and psychiatric disorders in 26 tissues using JTI analysis. Grey blocks indicate the association was unavailable. Blocks with numbers only indicate the unadjusted $P \geq 0.05$. Blocks with numbers and "*" indicate the unadjusted $P < 0.05$. Blocks with numbers and "**" indicate the Bonferroni-adjusted $P < 0.05$.

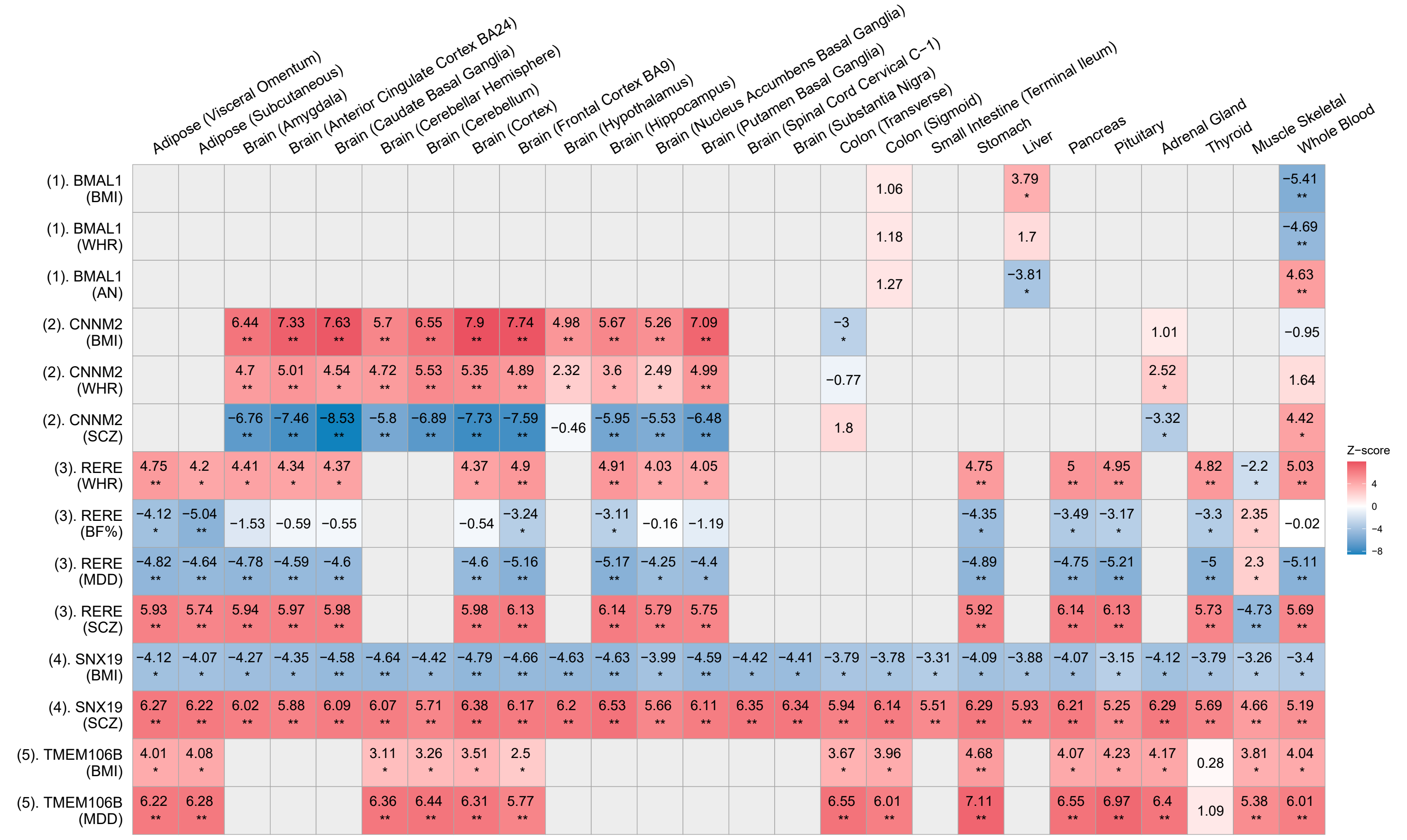
Adipose (Visceral Omentum)
Adipose (Subcutaneous)
Brain (Amygdala)
Brain (Anterior Cingulate Cortex BA24)
Brain (Caudate Basal Ganglia)
Brain (Cerebellar Hemisphere)
Brain (Cerebellum)
Brain (Cortex)
Brain (Frontal Cortex BA9)
Brain (Hypothalamus)
Brain (Hippocampus)
Brain (Nucleus Accumbens Basal Ganglia)
Brain (Putamen Basal Ganglia)
Brain (Spinal Cord Cervical C−1)
Brain (Substantia Nigra)
Colon (Transverse)
Colon (Sigmoid)
Small Intestine (Terminal Ileum)
Stomach
Liver
Pancreas
Pituitary
Adrenal Gland
Thyroid
Muscle Skeletal
Whole Blood
(1). BMAL1 (BMI): 1.06; 3.79 *; −5.41 **
(1). BMAL1 (WHR): 1.18; 1.7; −4.69 **
(1). BMAL1 (AN): 1.27; −3.81 *; 4.63 **
(2). CNNM2 (BMI): 6.44 **; 7.33 **; 7.63 **; 5.7 **; 6.55 **; 7.9 **; 7.74 **; 4.98 **; 5.67 **; 5.26 **; 7.09 **; −3 *; 1.01; −0.95
(2). CNNM2 (WHR): 4.7 **; 5.01 **; 4.54 *; 4.72 **; 5.53 **; 5.35 **; 4.89 **; 2.32 *; 3.6 *; 2.49 *; 4.99 **; −0.77; 2.52 *; 1.64
(2). CNNM2 (SCZ): −6.76 **; −7.46 **; −8.53 **; −5.8 **; −6.89 **; −7.73 **; −7.59 **; −0.46; −5.95 **; −5.53 **; −6.48 **; 1.8; −3.32 *; 4.42 *
(3). RERE (WHR): 4.75 **; 4.2 *; 4.41 *; 4.34 *; 4.37 *; 4.37 *; 4.9 **; 4.91 **; 4.03 *; 4.05 *; 4.75 **; 5 **; 4.95 **; 4.82 **; −2.2 *; 5.03 **
(3). RERE (BF%): −4.12 *; −5.04 **; −1.53; −0.59; −0.55; −0.54; −3.24 *; −3.11 *; −0.16; −1.19; −4.35 *; −3.49 *; −3.17 *; −3.3 *; 2.35 *; −0.02
(3). RERE (MDD): −4.82 **; −4.64 **; −4.78 **; −4.59 **; −4.6 **; −4.6 **; −5.16 **; −5.17 **; −4.25 *; −4.4 *; −4.89 **; −4.75 **; −5.21 **; −5 **; 2.3 *; −5.11 **
(3). RERE (SCZ): 5.93 **; 5.74 **; 5.94 **; 5.97 **; 5.98 **; 5.98 **; 6.13 **; 6.14 **; 5.79 **; 5.75 **; 5.92 **; 6.14 **; 6.13 **; 5.73 **; −4.73 **; 5.69 **
(4). SNX19 (BMI): −4.12 *; −4.07 *; −4.27 *; −4.35 *; −4.58 **; −4.64 **; −4.42 *; −4.79 **; −4.66 **; −4.63 **; −4.63 **; −3.99 *; −4.59 **; −4.42 *; −4.41 *; −3.79 *; −3.78 *; −3.31 *; −4.09 *; −3.88 *; −4.07 *; −3.15 *; −4.12 *; −3.79 *; −3.26 *; −3.4 *
(4). SNX19 (SCZ): 6.27 **; 6.22 **; 6.02 **; 5.88 **; 6.09 **; 6.07 **; 5.71 **; 6.38 **; 6.17 **; 6.2 **; 6.53 **; 5.66 **; 6.11 **; 6.35 **; 6.34 **; 5.94 **; 6.14 **; 5.51 **; 6.29 **; 5.93 **; 6.21 **; 5.25 **; 6.29 **; 5.69 **; 4.66 **; 5.19 **
(5). TMEM106B (BMI): 4.01 *; 4.08 *; 3.11 *; 3.26 *; 3.51 *; 2.5 *; 3.67 *; 3.96 *; 4.68 **; 4.07 *; 4.23 *; 4.17 *; 0.28; 3.81 *; 4.04 *
(5). TMEM106B (MDD): 6.22 **; 6.28 **; 6.36 **; 6.44 **; 6.31 **; 5.77 **; 6.55 **; 6.01 **; 7.11 **; 6.55 **; 6.97 **; 6.4 **; 1.09; 5.38 **; 6.01 **
Z−score
4
0
−4
−8

Supplementary Figure 6. Associations between genetically predicted expression of shared genes and adiposity traits and psychiatric disorders of general population in 26 tissues using GIFT analysis.

Grey blocks indicate the association was unavailable. Blocks with numbers only indicate the unadjusted $P \geq 0.05$. Blocks with numbers and "*" indicate the unadjusted $P < 0.05$. Blocks with numbers and "**" indicate the Bonferroni-adjusted $P < 0.05$.

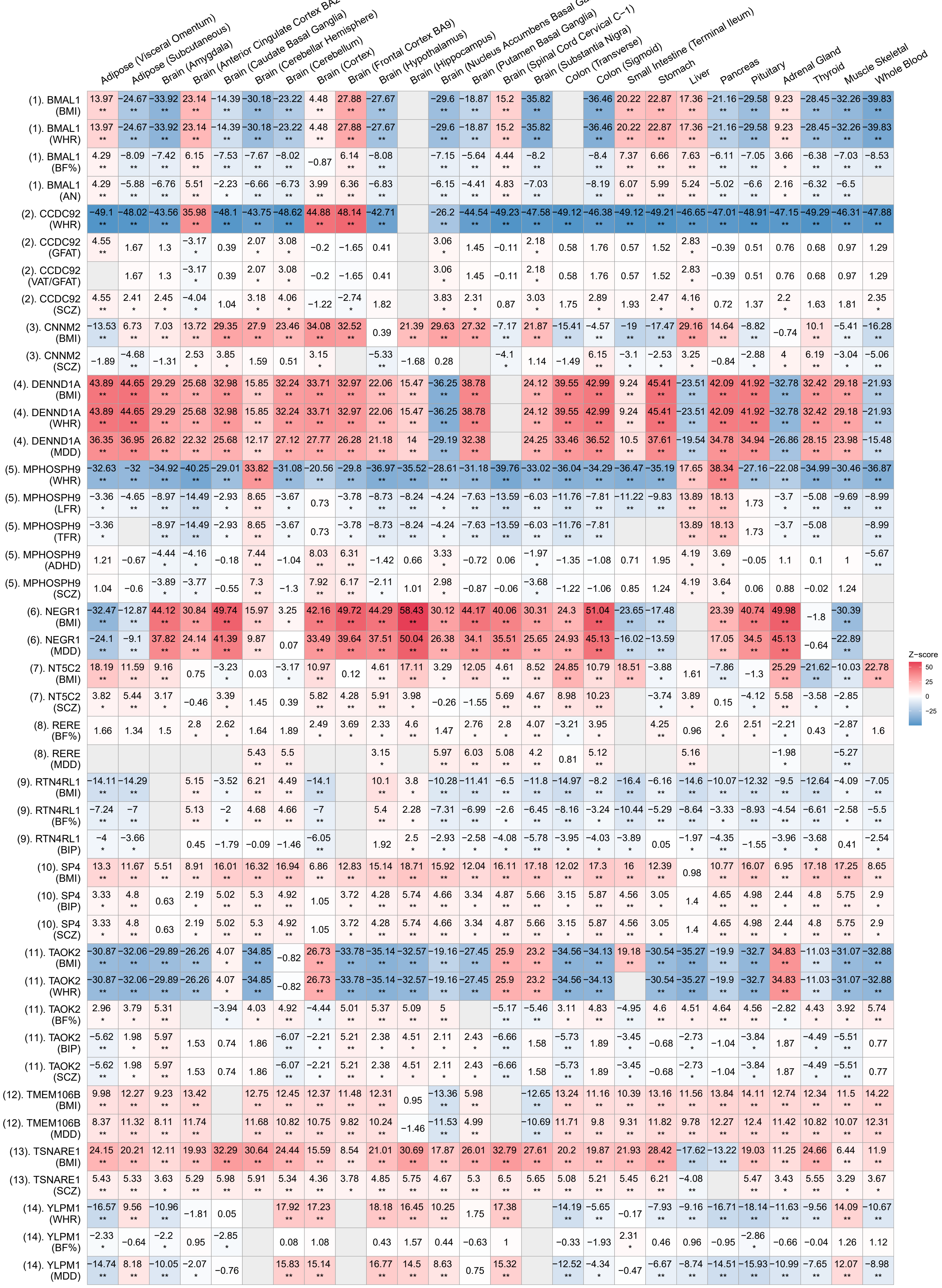
Adipose (Visceral Omentum)
Adipose (Subcutaneous)
Brain (Amygdala)
Brain (Anterior Cingulate Cortex BA24)
Brain (Caudate Basal Ganglia)
Brain (Cerebellar Hemisphere)
Brain (Cerebellum)
Brain (Cortex)
Brain (Frontal Cortex BA9)
Brain (Hypothalamus)
Brain (Hippocampus)
Brain (Nucleus Accumbens Basal Ganglia)
Brain (Putamen Basal Ganglia)
Brain (Spinal Cord Cervical C-1)
Brain (Substantia Nigra)
Colon (Transverse)
Colon (Sigmoid)
Small Intestine (Terminal Ileum)
Stomach
Liver
Pancreas
Pituitary
Adrenal Gland
Thyroid
Muscle Skeletal
Whole Blood
(1). BMAL1 (BMI)
(1). BMAL1 (WHR)
(1). BMAL1 (BF%)
(1). BMAL1 (AN)
(2). CCDC92 (WHR)
(2). CCDC92 (GFAT)
(2). CCDC92 (VAT/GFAT)
(2). CCDC92 (SCZ)
(3). CNNM2 (BMI)
(3). CNNM2 (SCZ)
(4). DENND1A (BMI)
(4). DENND1A (WHR)
(4). DENND1A (MDD)
(5). MPHOSPH9 (WHR)
(5). MPHOSPH9 (LFR)
(5). MPHOSPH9 (TFR)
(5). MPHOSPH9 (ADHD)
(5). MPHOSPH9 (SCZ)
(6). NEGR1 (BMI)
(6). NEGR1 (MDD)
(7). NT5C2 (BMI)
(7). NT5C2 (SCZ)
(8). RERE (BF%)
(8). RERE (MDD)
(9). RTN4RL1 (BMI)
(9). RTN4RL1 (BF%)
(9). RTN4RL1 (BIP)
(10). SP4 (BMI)
(10). SP4 (BIP)
(10). SP4 (SCZ)
(11). TAOK2 (BMI)
(11). TAOK2 (WHR)
(11). TAOK2 (BF%)
(11). TAOK2 (BIP)
(11). TAOK2 (SCZ)
(12). TMEM106B (BMI)
(12). TMEM106B (MDD)
(13). TSNARE1 (BMI)
(13). TSNARE1 (SCZ)
(14). YLPM1 (WHR)
(14). YLPM1 (BF%)
(14). YLPM1 (MDD)
Z-score
50
25
0
−25

Supplementary Figure 7. Associations between genetically predicted expression of shared genes and women's adiposity traits and psychiatric disorders in 26 tissues using GIFT analysis.

Grey blocks indicate the association was unavailable. Blocks with numbers only indicate the unadjusted $P \geq 0.05$. Blocks with numbers and "*" indicate the unadjusted $P < 0.05$. Blocks with numbers and "**" indicate the Bonferroni-adjusted $P < 0.05$.

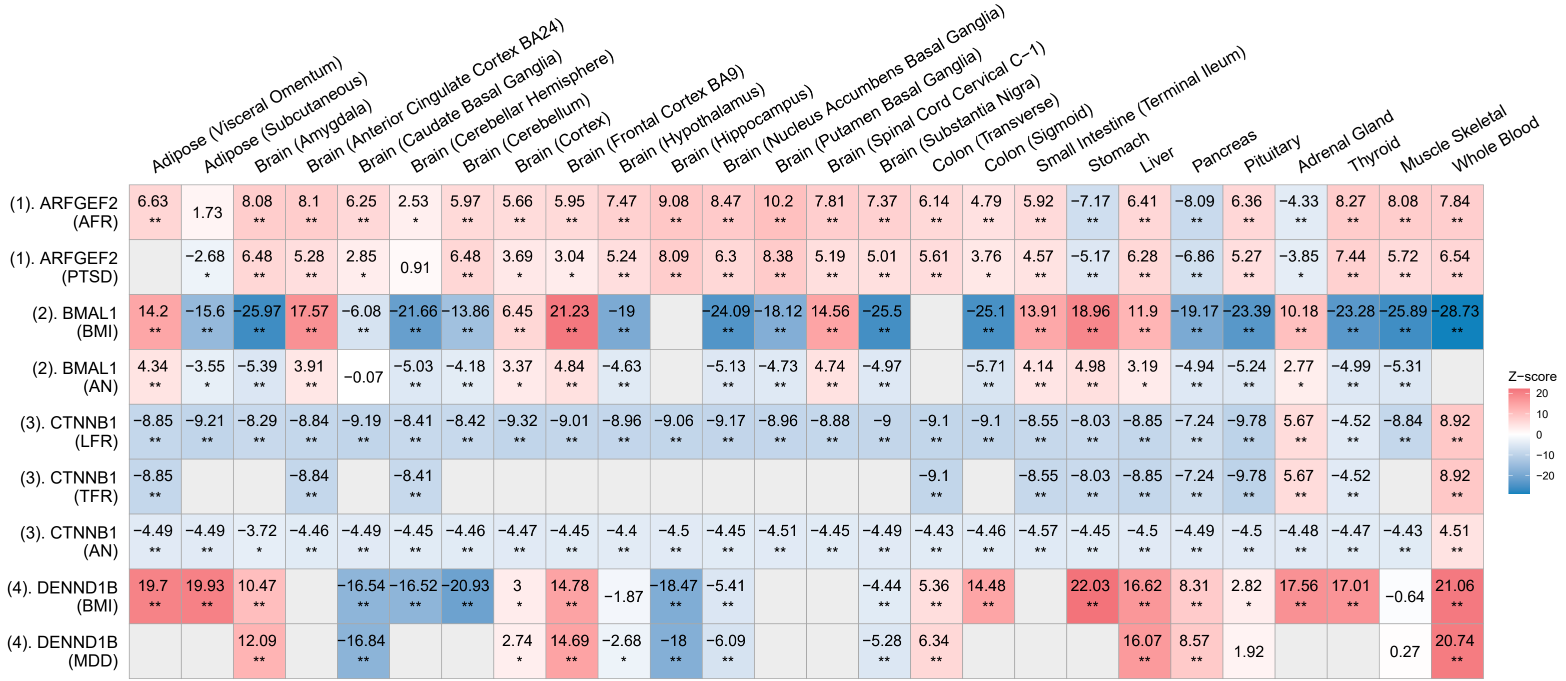
Adipose (Visceral Omentum)
Adipose (Subcutaneous)
Brain (Amygdala)
Brain (Anterior Cingulate Cortex BA24)
Brain (Caudate Basal Ganglia)
Brain (Cerebellar Hemisphere)
Brain (Cerebellum)
Brain (Cortex)
Brain (Frontal Cortex BA9)
Brain (Hypothalamus)
Brain (Hippocampus)
Brain (Nucleus Accumbens Basal Ganglia)
Brain (Putamen Basal Ganglia)
Brain (Spinal Cord Cervical C−1)
Brain (Substantia Nigra)
Colon (Transverse)
Colon (Sigmoid)
Small Intestine (Terminal Ileum)
Stomach
Liver
Pancreas
Pituitary
Adrenal Gland
Thyroid
Muscle Skeletal
Whole Blood
(1). ARFGEF2 (AFR) 6.63 ** 1.73 8.08 ** 8.1 ** 6.25 ** 2.53 * 5.97 ** 5.66 ** 5.95 ** 7.47 ** 9.08 ** 8.47 ** 10.2 ** 7.81 ** 7.37 ** 6.14 ** 4.79 ** 5.92 ** −7.17 ** 6.41 ** −8.09 ** 6.36 ** −4.33 ** 8.27 ** 8.08 ** 7.84 **
(1). ARFGEF2 (PTSD) −2.68 * 6.48 ** 5.28 ** 2.85 * 0.91 6.48 ** 3.69 * 3.04 * 5.24 ** 8.09 ** 6.3 ** 8.38 ** 5.19 ** 5.01 ** 5.61 ** 3.76 * 4.57 ** −5.17 ** 6.28 ** −6.86 ** 5.27 ** −3.85 * 7.44 ** 5.72 ** 6.54 **
(2). BMAL1 (BMI) 14.2 ** −15.6 ** −25.97 ** 17.57 ** −6.08 ** −21.66 ** −13.86 ** 6.45 ** 21.23 ** −19 ** −24.09 ** −18.12 ** 14.56 ** −25.5 ** −25.1 ** 13.91 ** 18.96 ** 11.9 ** −19.17 ** −23.39 ** 10.18 ** −23.28 ** −25.89 ** −28.73 **
(2). BMAL1 (AN) 4.34 ** −3.55 * −5.39 ** 3.91 ** −0.07 −5.03 ** −4.18 ** 3.37 * 4.84 ** −4.63 ** −5.13 ** −4.73 ** 4.74 ** −4.97 ** −5.71 ** 4.14 ** 4.98 ** 3.19 * −4.94 ** −5.24 ** 2.77 * −4.99 ** −5.31 **
(3). CTNNB1 (LFR) −8.85 ** −9.21 ** −8.29 ** −8.84 ** −9.19 ** −8.41 ** −8.42 ** −9.32 ** −9.01 ** −8.96 ** −9.06 ** −9.17 ** −8.96 ** −8.88 ** −9 ** −9.1 ** −9.1 ** −8.55 ** −8.03 ** −8.85 ** −7.24 ** −9.78 ** 5.67 ** −4.52 ** −8.84 ** 8.92 **
(3). CTNNB1 (TFR) −8.85 ** −8.84 ** −8.41 ** −9.1 ** −8.55 ** −8.03 ** −8.85 ** −7.24 ** −9.78 ** 5.67 ** −4.52 ** 8.92 **
(3). CTNNB1 (AN) −4.49 ** −4.49 ** −3.72 * −4.46 ** −4.49 ** −4.45 ** −4.46 ** −4.47 ** −4.45 ** −4.4 ** −4.5 ** −4.45 ** −4.51 ** −4.45 ** −4.49 ** −4.43 ** −4.46 ** −4.57 ** −4.45 ** −4.5 ** −4.49 ** −4.5 ** −4.48 ** −4.47 ** −4.43 ** 4.51 **
(4). DENND1B (BMI) 19.7 ** 19.93 ** 10.47 ** −16.54 ** −16.52 ** −20.93 ** 3 * 14.78 ** −1.87 −18.47 ** −5.41 ** −4.44 ** 5.36 ** 14.48 ** 22.03 ** 16.62 ** 8.31 ** 2.82 * 17.56 ** 17.01 ** −0.64 21.06 **
(4). DENND1B (MDD) 12.09 ** −16.84 ** 2.74 * 14.69 ** −2.68 * −18 ** −6.09 ** −5.28 ** 6.34 ** 16.07 ** 8.57 ** 1.92 0.27 20.74 **
Z−score
20
10
0
−10
−20

Supplementary Figure 8. Associations between genetically predicted expression of shared genes and men's adiposity traits and psychiatric disorders in 26 tissues using GIFT analysis.

Grey blocks indicate the association was unavailable. Blocks with numbers only indicate the unadjusted $P \geq 0.05$. Blocks with numbers and "*" indicate the unadjusted $P < 0.05$. Blocks with numbers and "**" indicate the Bonferroni-adjusted $P < 0.05$.

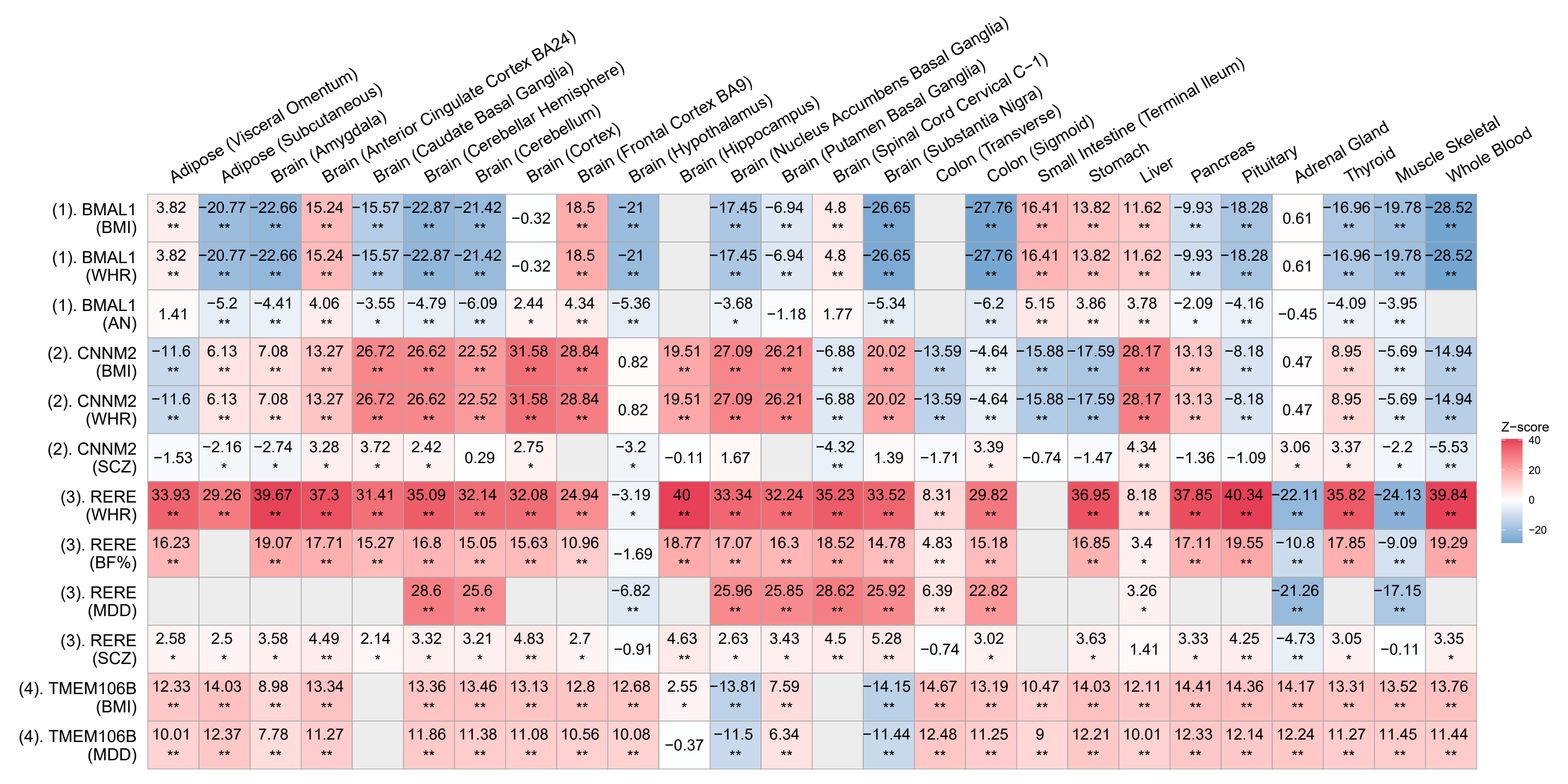
Adipose (Visceral Omentum)
Adipose (Subcutaneous)
Brain (Amygdala)
Brain (Anterior Cingulate Cortex BA24)
Brain (Caudate Basal Ganglia)
Brain (Cerebellar Hemisphere)
Brain (Cerebellum)
Brain (Cortex)
Brain (Frontal Cortex BA9)
Brain (Hypothalamus)
Brain (Hippocampus)
Brain (Nucleus Accumbens Basal Ganglia)
Brain (Putamen Basal Ganglia)
Brain (Spinal Cord Cervical C−1)
Brain (Substantia Nigra)
Colon (Transverse)
Colon (Sigmoid)
Small Intestine (Terminal Ileum)
Stomach
Liver
Pancreas
Pituitary
Adrenal Gland
Thyroid
Muscle Skeletal
Whole Blood
(1). BMAL1 (BMI) 3.82 ** −20.77 ** −22.66 ** 15.24 ** −15.57 ** −22.87 ** −21.42 ** −0.32 18.5 ** −21 ** −17.45 ** −6.94 ** 4.8 ** −26.65 ** −27.76 ** 16.41 ** 13.82 ** 11.62 ** −9.93 ** −18.28 ** 0.61 −16.96 ** −19.78 ** −28.52 **
(1). BMAL1 (WHR) 3.82 ** −20.77 ** −22.66 ** 15.24 ** −15.57 ** −22.87 ** −21.42 ** −0.32 18.5 ** −21 ** −17.45 ** −6.94 ** 4.8 ** −26.65 ** −27.76 ** 16.41 ** 13.82 ** 11.62 ** −9.93 ** −18.28 ** 0.61 −16.96 ** −19.78 ** −28.52 **
(1). BMAL1 (AN) 1.41 −5.2 ** −4.41 ** 4.06 ** −3.55 * −4.79 ** −6.09 ** 2.44 * 4.34 ** −5.36 ** −3.68 * −1.18 1.77 −5.34 ** −6.2 ** 5.15 ** 3.86 ** 3.78 ** −2.09 * −4.16 ** −0.45 −4.09 ** −3.95 **
(2). CNNM2 (BMI) −11.6 ** 6.13 ** 7.08 ** 13.27 ** 26.72 ** 26.62 ** 22.52 ** 31.58 ** 28.84 ** 0.82 19.51 ** 27.09 ** 26.21 ** −6.88 ** 20.02 ** −13.59 ** −4.64 ** −15.88 ** −17.59 ** 28.17 ** 13.13 ** −8.18 ** 0.47 8.95 ** −5.69 ** −14.94 **
(2). CNNM2 (WHR) −11.6 ** 6.13 ** 7.08 ** 13.27 ** 26.72 ** 26.62 ** 22.52 ** 31.58 ** 28.84 ** 0.82 19.51 ** 27.09 ** 26.21 ** −6.88 ** 20.02 ** −13.59 ** −4.64 ** −15.88 ** −17.59 ** 28.17 ** 13.13 ** −8.18 ** 0.47 8.95 ** −5.69 ** −14.94 **
(2). CNNM2 (SCZ) −1.53 −2.16 * −2.74 * 3.28 * 3.72 * 2.42 * 0.29 2.75 * −3.2 * −0.11 1.67 −4.32 ** 1.39 −1.71 3.39 * −0.74 −1.47 4.34 ** −1.36 −1.09 3.06 * 3.37 * −2.2 * −5.53 **
(3). RERE (WHR) 33.93 ** 29.26 ** 39.67 ** 37.3 ** 31.41 ** 35.09 ** 32.14 ** 32.08 ** 24.94 ** −3.19 * 40 ** 33.34 ** 32.24 ** 35.23 ** 33.52 ** 8.31 ** 29.82 ** 36.95 ** 8.18 ** 37.85 ** 40.34 ** −22.11 ** 35.82 ** −24.13 ** 39.84 **
(3). RERE (BF%) 16.23 ** 19.07 ** 17.71 ** 15.27 ** 16.8 ** 15.05 ** 15.63 ** 10.96 ** −1.69 18.77 ** 17.07 ** 16.3 ** 18.52 ** 14.78 ** 4.83 ** 15.18 ** 16.85 ** 3.4 * 17.11 ** 19.55 ** −10.8 ** 17.85 ** −9.09 ** 19.29 **
(3). RERE (MDD) 28.6 ** 25.6 ** −6.82 ** 25.96 ** 25.85 ** 28.62 ** 25.92 ** 6.39 ** 22.82 ** 3.26 * −21.26 ** −17.15 **
(3). RERE (SCZ) 2.58 * 2.5 * 3.58 * 4.49 ** 2.14 * 3.32 * 3.21 * 4.83 ** 2.7 * −0.91 4.63 ** 2.63 * 3.43 * 4.5 ** 5.28 ** −0.74 3.02 * 3.63 * 1.41 3.33 * 4.25 ** −4.73 ** 3.05 * −0.11 3.35 *
(4). TMEM106B (BMI) 12.33 ** 14.03 ** 8.98 ** 13.34 ** 13.36 ** 13.46 ** 13.13 ** 12.8 ** 12.68 ** 2.55 * −13.81 ** 7.59 ** −14.15 ** 14.67 ** 13.19 ** 10.47 ** 14.03 ** 12.11 ** 14.41 ** 14.36 ** 14.17 ** 13.31 ** 13.52 ** 13.76 **
(4). TMEM106B (MDD) 10.01 ** 12.37 ** 7.78 ** 11.27 ** 11.86 ** 11.38 ** 11.08 ** 10.56 ** 10.08 ** −0.37 −11.5 ** 6.34 ** −11.44 ** 12.48 ** 11.25 ** 9 ** 12.21 ** 10.01 ** 12.33 ** 12.14 ** 12.24 ** 11.27 ** 11.45 ** 11.44 **
Z−score
40
20
0
−20
